# Integrated Transcriptome and Metabolomics Analyses Show MYC as a Potential Therapeutic Target for Behçet's Uveitis

**DOI:** 10.1002/advs.202417843

**Published:** 2025-06-30

**Authors:** He Li, Lei Zhu, Zhaohuai Li, Yihan Zhang, Genxian Zhang, Xuening Peng, Dongting Wu, Qi Jiang, Rong Wang, Renbing Jia, Song Guo Zheng, Wenru Su

**Affiliations:** ^1^ Department of Ophthalmology Ninth People's Hospital Shanghai Jiao Tong University School of Medicine Shanghai 200011 China; ^2^ State Key Laboratory of Eye Health Department of Ophthalmology Shanghai Ninth People's Hospital Shanghai Jiao Tong University School of Medicine Shanghai 200001 China; ^3^ Division of Rheumatology and Immunology Department of Immunology School of Cell and Gene Therapy Songjiang Research Institute Shanghai Songjiang District Central Hospital Shanghai Jiaotong University School of Medicine Shanghai 200011 China; ^4^ State Key Laboratory of Ophthalmology Zhongshan Ophthalmic Center Sun Yat‐sen University Guangdong Provincial Key Laboratory of Ophthalmology and Visual Science Guangzhou 510060 China

**Keywords:** Behçet's uveitis, experimental autoimmune uveitis, glycolysis, metabolomics, single‐cell RNA sequencing

## Abstract

Behçet's uveitis (BU), characterized by recurrent bilateral panuveitis, is a severe manifestation of Behcet's disease (BD). However, disease‐specific metabolic alterations in BU remain largely unknown. Here, untargeted metabolomics and single‐cell RNA sequencing (scRNA‐seq) are performed in patients with BU and healthy controls (HC). scRNA‐seq data of experimental autoimmune uveitis (EAU) mice are also incorporated. The data showed an altered metabolic profile, characterized by upregulated glycolysis in BU. MYC is predicted to be a hub molecule regulating glycolysis and T cell response. Notably, it is discovered that the expression level of MYC is higher in BU compare to HC and may reflect the treatment response of BU disease. Correspondingly, the scRNA‐seq data of EAU mice also reveal higher glycolysis levels and MYC expression. Further studies reveal that inhibition of MYC repressed glycolysis and exerted therapeutic effects similar to those of glycolysis inhibitors, including amelioration of EAU and repression of the abnormal response of effector T cells (T helper [Th]‐1 and Th17 cells). Mechanically, inhibiting MYC disrupts the glycolysis‐PI3K signaling circuit to curb the effector T cell response in uveitis. Collectively, the study indicated that MYC promoted glycolysis to fuel abnormal T‐cell responses, thus therapeutically targeting MYC would provide an attractive approach for treating BU.

## Graphical abstract

Integrated single‐cell transcriptomic and metabolomic analyses reveal that MYC‐driven glycolysis activates the PI3K‐AKT‐FOXO1 pathway, disrupting Th17/Treg balance and promoting uveitis. MYC inhibition restores immune homeostasis and alleviates disease symptoms. These findings highlight MYC as a key metabolic regulator and suggest its potential as a therapeutic target in Behçet's uveitis.

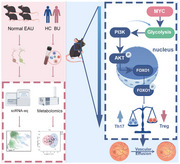

## 1 Introduction

Behçet' uveitis (BU), characterized by recurrent bilateral nongranulomatous pan‐uveitis, is one of the most serious manifestations of Behçet disease (BD) because of its high rate of causing blindness.^[^ ^1^ ^]^ BD is a multisystem, chronic, relapsing disease whose pathogenesis is associated with autoimmune and autoinflammatory factors.^[^ ^2^, ^3^, ^4^ ^]^ The major BD symptoms include oral ulcers, genital ulcerations, skin lesions, and ocular involvement.^[^ ^5^ ^]^ BU occurs in 60–80% of patients with BD^[^ ^4^ ^]^ and is one of the most common types of uveitis in Asia.^[^ ^6^ ^]^ Although the clinical features and diagnostic criteria are well described, the pathogenesis of BU disease remains unclear. Current treatment of this disease is largely untargeted. Systemic corticosteroids and immunosuppressive drugs are first‐line treatments for patients with BU.^[^ ^7^ ^]^ However, some patients respond inadequately to these agents and some even experience intolerable side effects.^[^ ^7^ ^]^ Moreover, there are currently no reliable disease‐specific biomarkers to evaluate different disease stages and predict treatment responses. Therefore, knowledge of the pathogenesis of BU is needed to develop novel, effective, and targeted therapies.

Recent studies have shown the role of immune cells in the pathogenesis of BU. Enhanced effector T cells (T helper [Th]‐1 and Th17 cells) and suppressed regulatory T (Treg) cells have been observed in patients with BU.^[^ ^8^, ^9^ ^]^ Elevated IFN‐𝛾, IL‐17, and TNF‐𝛼 levels also occur in this group of patients.^[^ ^4^ ^]^ Experimental autoimmune uveitis (EAU) is the classical animal model of uveitis,^[^ ^10^ ^]^ which was established by the autoantigen, interphotoreceptor retinoid‐binding protein 1–20 (IRBP1‐20).^[^ ^10^ ^]^ Studies on EAU mice indicated that effector T cells (particularly Th1 and Th17 cells) dominated in uveitis pathogenesis, and inhibition of these cells could reduce the EAU symptom.^[^ ^11^, ^12^ ^]^ Therefore, investigating the regulatory mechanisms of T cells may provide novel therapeutic targets for BU treatment.

In recent years, the importance of cellular metabolism in immune cells and autoimmune diseases has been proposed, thus initiating a novel field named immunometabolism.^[^ ^13^ ^]^ Immune cells require metabolic reprogramming to attain their energetic and biomolecular needs in response to challenges, including pathogens or autoantigens.^[^ ^14^ ^]^ Regarding these challenges, naïve T cells are activated and differentiate into effector T cells, during which glycolysis increases to meet the energy demands.^[^ ^15^ ^]^ The dysregulation of metabolic programs is associated with immune‐mediated diseases, including systemic lupus erythematosus^[^ ^13^ ^]^ and rheumatoid arthritis.^[^ ^16^ ^]^ During BU, key molecules that affect immunometabolism and induce abnormal immune cell responses are unknown.

Single‐cell RNA sequencing (scRNA‐seq) allows high‐throughput and high‐resolution analysis of individual cells^[^ ^17^ ^]^ and has unprecedented value for discovering the cellular metabolic states of immune cells.^[^ ^18^, ^19^ ^]^ Metabolomics studies have explored endogenous metabolites of cellular processes.^[^ ^20^ ^]^ This study combined untargeted metabolomics and single‐cell transcriptomic data to characterize changes in gene expression and metabolism during BU. Metabolic alterations during BU were characterized by upregulated glycolysis. Subsequent transcriptional analysis predicted that MYC may be a hub molecule that regulates the metabolic state of T cells during BU. CD4+ T effector memory cells (CD4+Tem) increased the most in glycolysis and MYC expression. Notably, MYC expression was higher during BU and correlated with disease response to treatment. Meanwhile, in EAU mice, MYC inhibitors ameliorated EAU symptoms and repressed Th1 and Th17 cells. Mechanistically, inhibition of MYC disrupted the positive feedback circuit between glycolysis and the PI3K‐AKT‐FOXO1 pathway to curb the abnormal T cell response. Thus, MYC might be a potential therapeutic target for BU because of its glycolysis regulatory capacity.

## 2 Results

### 2.1 Study Design and Serum Metabolic Profile of BU

**Figure 1:**
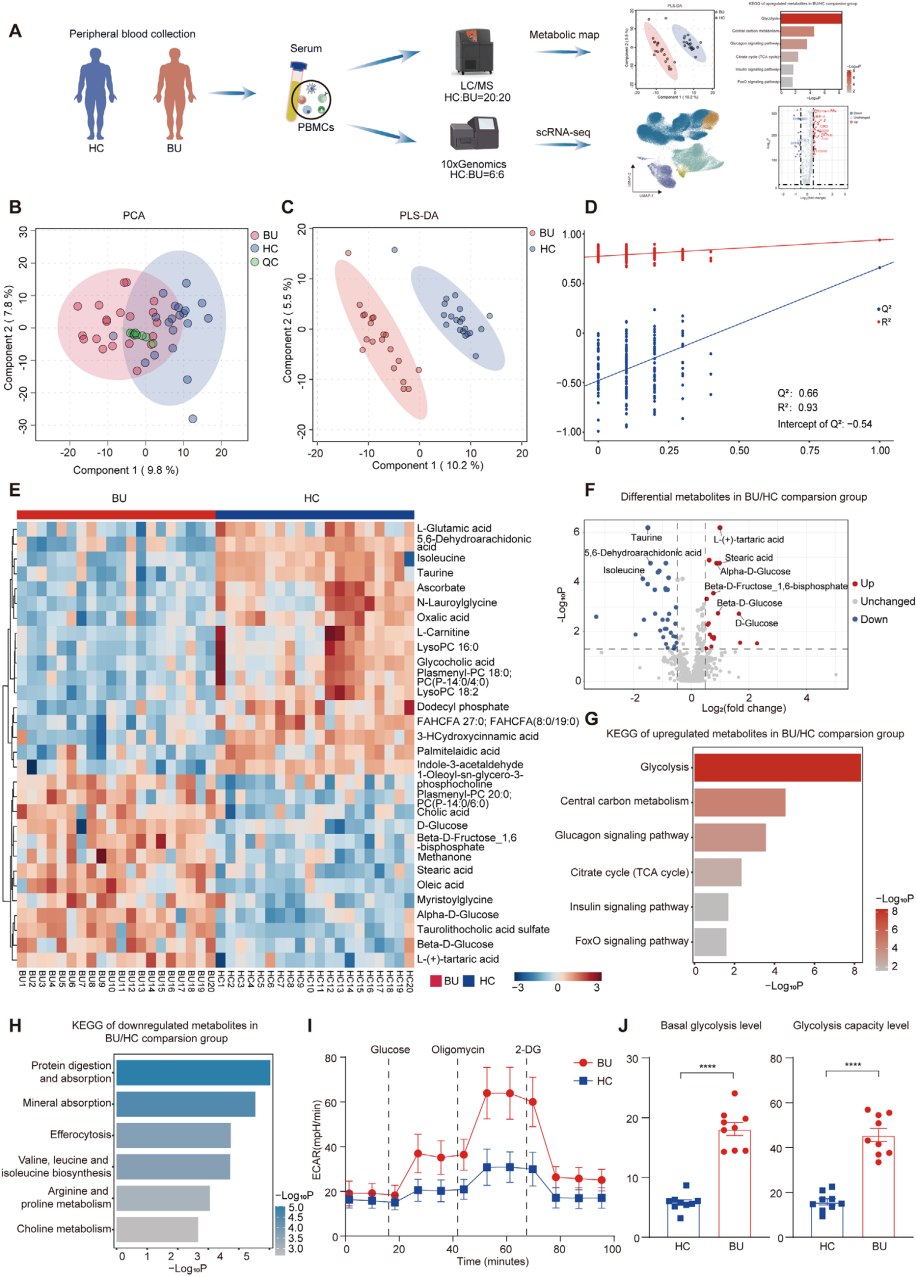
Patients with BU showed a different metabolic profile compared to HCs. A) Schematic of the experimental design. B) PCA plot from patients with BU, HC and QC groups. C) PLS‐DA plot from patients with BU and HCs. D) The validation of the PLS‐DA models from patients with BU and HCs. E) The top 30 significantly altered metabolites between serum from patients with BU and HCs. F) Volcano plot shows the significantly altered metabolites between patients with BU and HCs. Red and blue dots indicate upregulated and downregulated metabolites in patients with BU compared to HCs. x axis: Fold change. y axis: P‐values with FDR adjustment. G) Bar plot shows KEGG of upregulated metabolites between patients with BU and HCs. The color represents ‐Log10 of enrichment P value, with red being high and gray being low. x axis: enrichment P values. H) Bar plot shows KEGG of downregulated metabolites between patients with BU and HCs. The color represents ‐Log10 of enrichment P value, with blue being high and gray being low. x axis: enrichment P values. I,J) The basal glycolysis level and glycolysis capacity level of PBMCs from patients with BU and HCs were measured by glycolysis stress assay. Each group contains nine samples. Data expressed as mean ± SEM. Significance was determined using unpaired two‐tailed student's t test. ^****^ *p* <0.0001. ECAR: extracelluar acidity rate; 2‐DG: 2‐deoxy‐glucose.

To explore the metabolic and transcriptional alterations during BU, we conducted untargeted metabolic analysis and scRNA‐seq on the peripheral blood mononuclear cells (PBMCs) from healthy controls (HCs) and patients with BU (**Figure** **1**A). In the process of metabolic analysis between 20 BU and 20 HC samples, a total of 577 level 2 metabolites were captured and identified according to the classification proposed by the Metabolomics Standards Initiative.^[^ ^21^ ^]^ We performed Principal component analysis (PCA) on the pre‐processed data and found group differences between BU and HC groups (Figure 1B). Additionally, the PCA results indicated minimal batch effects, as the QC samples were consistently clustered together, showing that the analysis was stable across the entire testing period (Figure 1B). To highlight the differences between groups, Partial least squares discriminant analysis (PLS‐DA) was performed to visualize the differences in metabolic profiles between patients with BU and HCs (Figure 1C). A clear difference was observed between patients with BU and HCs based on the altered metabolites (Figure 1C). In our PLS‐DA model, R2 was 0.93, indicating that 93% of the variance in the data was explained by the model. The Q2 value of 0.66 suggests that the model has adequate predictive ability (Figure 1D). Among the altered metabolites, 18 metabolites were up‐regulated, and 35 metabolites were down‐regulated in BU compared to HC. The top 30 significantly altered metabolites are shown in Figure 1E. The volcano plot showed that alpha‐D‐Glucose and D‐Glucose were upregulated in BU, while taurine and isoleucine were downregulated (Figure 1F). To further identify the functions of the altered metabolites in BU, we analyzed the Kyoto Encyclopedia of Genes and Genomes metabolic library using MetaboAnalyst (Figure 1G). We observed that pathways annotated as “Glycolysis,” “Insulin signaling pathway,” and “FOXO signaling pathway” were significantly upregulated in BU (Figure 1G). These significantly upregulated pathways are highly correlated with glycolysis.^[^ ^20^, ^22^, ^23^, ^24^ ^]^ Insulin regulates glucose metabolism including the activity of glycolysis.^[^ ^24^ ^]^ FOXO signaling is regulated by glycolysis via the PI3K‐AKT pathway.^[^ ^22^, ^23^ ^]^ These results indicated a metabolic switch to glycolysis in BU. Highly active glycolysis usually occurs in autoimmune conditions to support rapid energy consumption in the differentiation and effector function of some immune cells, such as the effector T cells.^[^ ^18^, ^25^ ^]^ These interrelated pathways indicated an important role of glycolysis in BU. In addition, pathways related to valine, leucine, and isoleucine biosynthesis were downregulated (Figure 1H). To validate the glycolytic activity in BU, a seahorse extracellular flux analyzer was used to measure the glycolytic activity of PBMCs derived from patients with BU and HCs by monitoring the extracellular acidification rate (ECAR). The results showed that the basal glycolysis and glycolytic capacity of PBMC from patients with BU were higher than those of HCs (Figure 1I,J). To further determine whether this metabolic alteration is intrinsic to T cells, we isolated CD4+ T cells and performed the same ECAR assay. Consistently, BU‐derived T cells exhibited significantly enhanced glycolysis activity (Figure S1A,B, Supporting Information). These results indicate that patients with BU have altered serum metabolic profiles compared with HCs, including a significant increase in glycolysis.

### 2.2 scRNA‐Seq Analysis of PBMC from Patients with BU

**Figure 2:**
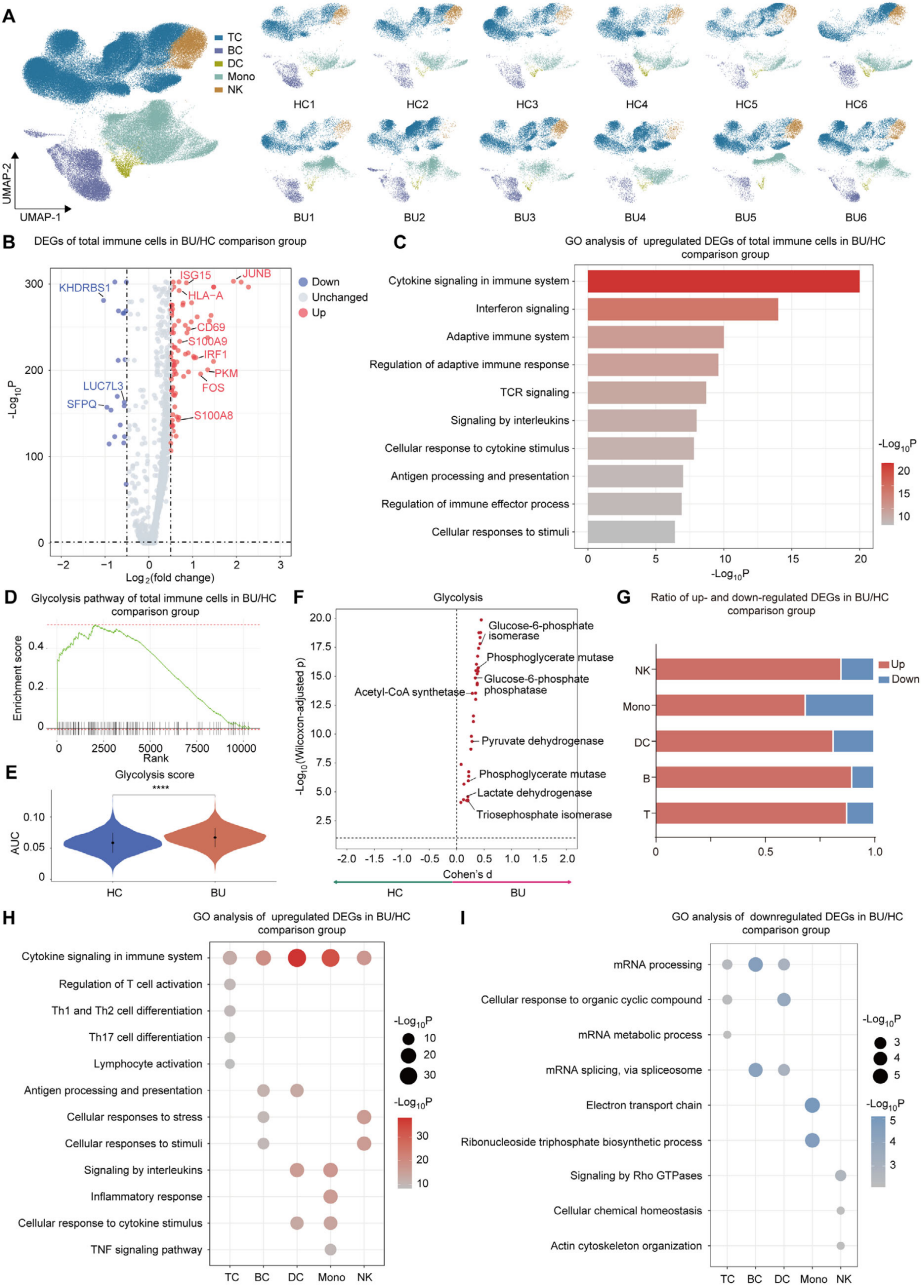
scRNA‐seq depicted altered transcriptome of PBMCs during BU A) UMAP clustering of PBMCs from all participants. B) Volcano plot shows DEGs of PBMCs between patients with BU and HCs. Red and blue dots indicate upregulated and downregulated DEGs in patients with BU compared to HCs. x axis: Fold change. y axis: P‐values with FDR adjustment. C) Bar plot shows representative GO terms enriched by the upregulated DEGs of PBMCs from patients with BU compared HCs. The color represents ‐Log10 of enrichment P value, with red being high and gray being low. x axis: enrichment P values. D) GSEA of DEGs in PBMCs from patients with BU compared to HCs shows the enrichment of glycolysis pathway. E) Violin plot shows AUcell score of glycolysis in PBMCs from patients with BU and HCs. Significance was determined using wilcoxon test. ^****^ *p* <.0001. F)Compass‐score glycolysis activity in PBMCs from patients with BU and HCs. G) The ratio of up‐ and ‐down regulated DEGs in each major cell type. H) Bubble plot shows representative GO terms enriched by the upregulated DEGs of each major cell type from patients with BU compared to HCs. The circle size and color represent ‐Log10 of enrichment P value, with red being high and gray being low. I) Bubble plot shows representative GO terms enriched by the downregulated DEGs of each major cell type from patients with BU compared to HCs. The circle size and color represent ‐Log10 of enrichment P value, with blue being high and gray being low.

To identify the transcriptional signature of BU disease, PBMCs were isolated from six patients with BU and six HCs and analyzed using scRNA‐seq. T cell (TC: CD3D, IL7R), natural killer cell (NK: NCAM1), B cell (BC: CD79A, MS4A1), monocyte (MC: CD14, LYZ), and dendritic cell (DC: CLEC10A, CD1C) were annotated based on the expression of canonical lineage markers and other genes specifically upregulated in each cluster (**Figure** **2**A; Figure S1C,D, Supporting Information), as our previous paper pertaining to uveitis diseases.^[^ ^26^, ^27^, ^28^ ^]^ We used samples from six patients with BU and samples from six HCs as two groups (referred to as the BU group and HC group) for comparative analysis. To understand the global transcriptional changes during BU, we conducted differentially expressed gene (DEG) analysis between all immune cells from the BU group and those from the HC group at the single‐cell level. Genes involved in interleukin (IL)‐17 signaling (JUNB), interferon signaling (ISG15 and IRF1), immune cell activation (CD69), antigen processing and presentation (HLA‐A), inflammation (S100A9, FOS, and S100A8), and glycolysis (PKM) were upregulated during BU (Figure 2B). Genes involved in mRNA processing (KHDRBS1, LUC7L3, and SFPQ) were downregulated in BU (Figure 2B). We then performed Gene Ontology (GO) analysis to annotate the functions of these genes. Pathways, including “Cytokine signaling in the immune system,” “Interferon signaling,” “Adaptive immune system,” “TCR signaling” and “Signaling by interleukins” were upregulated, indicating enhanced adaptive immunity and T cell response during BU (Figure 2C). The downregulated pathways in the BU group were related to RNA processing (Figure S1E, Supporting Information).

To investigate whether the enhanced glycolysis exists in scRNA‐seq data of patients with BU, we performed gene set enrichment analysis (GSEA) of genes differentially expressed in PBMC from patients with BU and HCs. We obtained results similar to the GO analysis that the pathways related to inflammation, immune response, and cell activation were enhanced in the BU group (Figure S1E, Supporting Information). Additionally, the glycolysis pathway was enriched in the BU group (Figure S1E, Supporting Information; Figure 2D). Then, we used AUCell^[^ ^29^, ^30^ ^]^ to score the glycolysis pathway and observed that the Area Under the Curve (AUC) score for this pathway was higher in the BU group than in the HC group (Figure 2E). Furtherly, we used Compass to infer changes in cellular metabolic states based on scRNA‐seq data^[^ ^18^ ^]^ and found that the glycolytic reactions were more active in BU (Figure 2F). These results showed an increase in glycolysis during the BU.

Next, we conducted DEG analyses for each major immune cell type. The genetic changes of all five major immune cell types were mainly upregulated in BU (Figure 2G). GO analysis of the upregulated DEGs indicated enhanced antigen processing and presentation in B and dendritic cells (Figure 2H). Inflammation‐ and cytokine‐related pathways were enriched in dendritic cells and monocytes (Figure 2H). In T cells, pathways annotated as “regulation of T cell activation,” “Th1 and Th17 cell differentiation” and “Th17 cell differentiation” were upregulated (Figure 2H). These pathways are closely associated with BU.^[^ ^8^, ^9^ ^]^ GO analysis of the downregulated DEGs revealed that RNA processing‐related pathways were downregulated in B, dendritic, and T cells (Figure 2I). And, the ribonucleoside triphosphate biosynthesis‐related pathway was predicted to be downregulated in monocytes (Figure 2I). In NK cells, pathways related to cellular chemical homeostasis were predicted to be downregulated in BU (Figure 2I). Therefore, we observed that genes promoting inflammation and autoimmunity were upregulated in the PBMC of patients with BU. Moreover, enhanced glycolysis in BU was predicted using metabolic analysis tools during scRNA‐seq analysis.

### 2.3 MYC is a Potential Regulator of Glycolysis in T Cells during BU Pathogenesis

**Figure 3:**
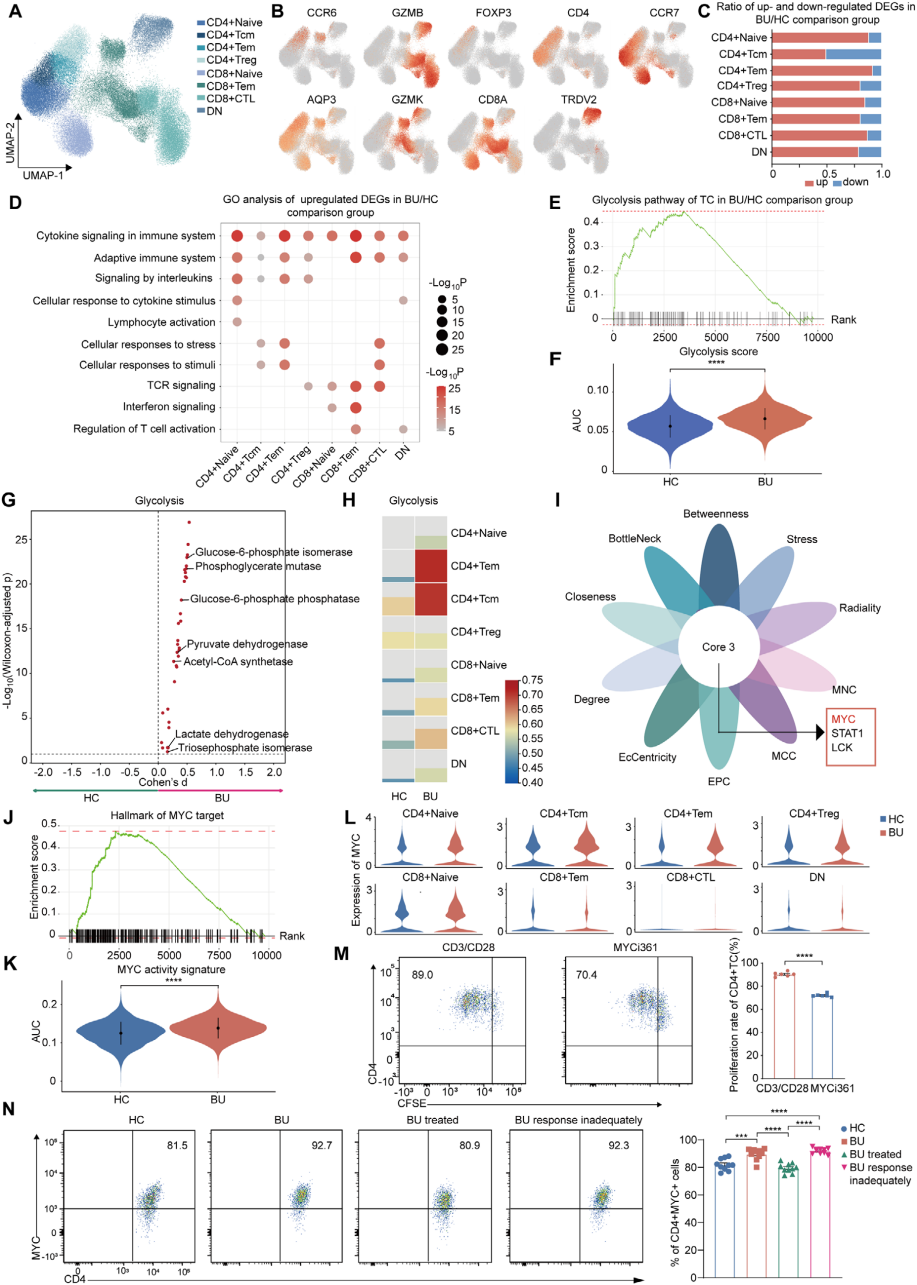
scRNA‐seq depicted altered transcriptome of T cells during BU A) UMAP clustering of T cells from all participants. B) Feature plots shows the expression of classic markers in each T cell subsets. C) The ratio of up‐ and ‐down regulated DEGs in each T cell subsets. D) Bubble plot shows representative GO terms enriched by the upregulated DEGs of each T cell subset from patients with BU compared to HCs. The circle size and color represent ‐Log10 of enrichment P value, with red being high and gray being low. E) GSEA of DEGs in T cells from patients with BU compared to HCs shows the enrichment of glycolysis pathway. F) Violin plot shows AUcell score of glycolysis in T cells from patients with BU and HCs. Significance was determined using wilcoxon test. ^****^ *p* <0.0001. G) Compass‐score glycolysis activity in T cells from patients with BU and HCs. H) Heatmap shows the scores of glycolysis each T cell subset from patients with BU compared to HCs using scMetabolism. I) Venn plot shows the six common hub genes predicted by 10 algorithms. Each algorithm calculates top 20 hub genes. J) GSEA of DEGs in T cells from patients with BU compared to HCs shows the enrichment of MYC tagets. K) Violin plot shows AUcell score of MYC activity in T cells from patients with BU and HCs. L) Violin plots shows the expression of MYC in each T cell subset in patients with BU and HCs. M) The proliferating rate of CD4+ T cells. Data represented as mean ± SEM from six independent experiments. Significance was determined using unpaired two‐tailed student's t test. ^****^ *p* <0.0001. N) The percentage of MYC+ cells in CD4+ T cells. Each group contains 10 samples. Data expressed as mean ± SEM. Significance was determined using two‐way ANOVA. ^***^ *p* <0.001, ^****^ *p* <0.0001.

Although abnormalities in various immune cells are critical contributors to autoimmune destruction in uveitis, T cells, especially effector T cells (e.g., Th1 and Th17 cells) and Treg are considered pivotal in the pathogenesis of uveitis and its classic animal model, EAU.^[^ ^31^, ^32^ ^]^ Solely transfer of autoreactive CD4+ T cells can induce EAU in naïve mice,^[^ ^33^, ^34^ ^]^ a capability not possessed by other cell types. Additionally, significantly upregulated glycolysis was observed in BU (Figure 1F,I). Increased glycolysis is a hallmark of T cell activation and is thought to be required to meet the metabolic demands of T cell differentiation and effector functions.^[^ ^35^, ^36^ ^]^ Thus, we then conducted a more in‐depth analysis focusing on T cells. We re‐clustered T cells and identified eight T cell subsets, including CD4+ naive T cells (CD4+ Navie), CD4+ central memory T cells (CD4+Tcm), CD4+ effector memory T cells (CD4+Tem), CD4+ Treg cells, CD8+ naive T cells, CD8+ effector memory T cells (CD8+Tem), CD8+ cytotoxic T lymphocytes (CD8+CTL), and double‐negative T cells (DN) (**Figure** **3**A,B; Figure S1G, Supporting Information). Double‐negative T cells were characterized by the expression of the T cell marker CD3 and were negative for CD4 and CD8, as previously reported.^[^ ^37^ ^]^ DEG analysis showed that the BU‐induced‐gene expression changes in T‐cell subsets were mostly upregulated (Figure 3C). GO analysis of the upregulated DEGs predicted that cytokine signaling, T cell activation, and adaptive immune responses were upregulated in nearly all T cell subsets (Figure 3D). Downregulated DEGs were enriched in pathways related to RNA processing in most T cell subsets (Figure S1H, Supporting Information).

We further explored the glycolytic activity of the T cells. The GSEA of genes differentially expressed in T cells from patients with BU and HCs indicated an enhanced glycolysis pathway during BU (Figure 3E). The AUC score of this pathway was higher in the BU group (Figure 3F). Additionally, the AUC scores of pathways related to PI3K‐AKT signaling and FOXO signaling were higher in the BU group (Figure S1I,J, Supporting Information). The compass algorithm also predicted an increased expression of glycolytic enzymes and higher glycolytic activity of T cells in BU (Figure 3G). Furthermore, we compared the glycolytic activity among T cell subsets using scMetabolism which was used to infer changes in metabolic pathways based on scRNA‐seq data.^[^ ^19^ ^]^ We found that the activity of the glycolysis pathway was increased in T cell subsets except for CD4+ Treg cells (Figure 3H). Among these cells, glycolytic activity of CD4+ Tem cell increased the most (Figure 3H).

To identify the molecular mechanism underlying the abnormal T cell response and highly active glycolysis in T cells during the BU process, we constructed a protein‐protein interaction network based on the upregulated DEGs in T cells from patients with BU (Figure S2, Supporting Information). Genes were scored, and hub genes were predicted using the CytoHubba plug‐in for Cytoscape^[^ ^38^, ^39^ ^]^ (Figure S2A, Supporting Information). The intersection of the top 20 hub genes calculated using 10 algorithms revealed three common genes: MYC, STAT1, and LCK (Figure 3I; Figure S2B, and Table S3, Supporting Information). MYC, is an essential regulator of glycolysis by controlling the expression of genes encoding glycolytic enzymes.^[^ ^40^ ^]^ Compared to MYC, the studies on the regulation of glycolysis by STAT1 and LCK are much less, even though STAT1 and LCK have been reported to be associated with uveitis.^[^ ^41^, ^42^ ^]^ Meanwhile, the GSEA of genes differentially expressed in T cells from patients with BU and HCs indicated enhanced MYC targets (Figure 3J). The AUC score of MYC activity was higher in the BU (Figure 3K). Therefore, we hypothesized that upregulated MYC expression correlates highly with enhanced glycolysis. Furthermore, we found that in BU, CD4+ T cell subsets and CD8+ Naïve T cells expressed relatively high levels of MYC (Figure 3L). Additionally, we have downloaded the scRNA‐seq data of BD without uveitis from a published study and re‐analyzed it along with our own data^[^ ^43^ ^]^ (Figure S3, Supporting Information). To ensure comparability, both cohorts included treatment‐naïve patients at initial disease onset, and the BD without uveitis group was matched to BU patients in terms of disease severity and comorbidities. The AUC scores of the glycolysis pathway in both total peripheral immune cells and T cells were highest in patients with BU, followed by BD without uveitis, and lowest in the HC group (Figure S3C,F, Supporting Information). Meanwhile, the MYC expression in T cells from patients with BU was upregulated in nearly all T cell subsets compared to BD without uveitis and the HC group (Figure S3G, Supporting Information). These results indicate that abnormally enhanced MYC expression and glycolysis might play an important role in BU.

To further explore the role of MYC in BU, we treated CD4+ T cells from BU with MYCi361, a MYC inhibitor. MYCi361 disturbed CD4+ T cell proliferation (Figure 3M). Moreover, we conducted flow cytometry and found that MYC expression was higher in CD4+ T cells from patients with BU than in those from HCs, and markedly decreased after treatment (Figure 3N). Additionally, MYC expression was significantly higher in patients who responded inadequately to corticosteroids and immunosuppressive drugs (Figure 3N). These results suggested that MYC expression might reflect the treatment response in BU disease.

### 2.4 Enhanced Glycolysis and MYC Expression were Observed in EAU

**Figure 4:**
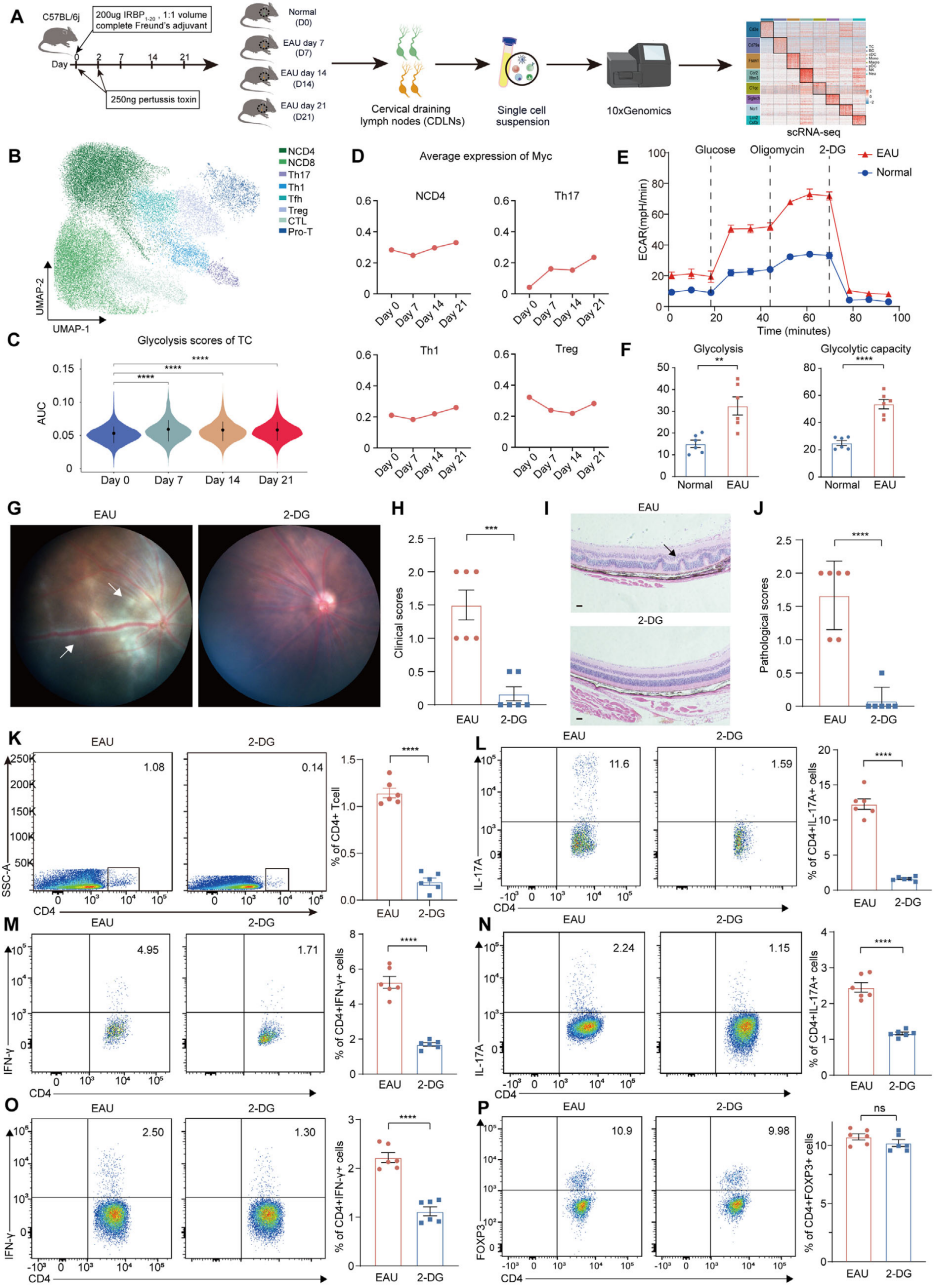
scRNA‐seq of EAU mice shows higher glycolysis and MYC expression A) Schematic of the experimental design. B) UMAP clustering of T cell. C) Violin plot shows AUcell score of glycolysis in T cells from mice on day0 (normal mice), day 7, day 14, and day 21 of EAU modeling. Significance was determined using wilcoxon test. ^****^ *p* <0.0001. D) Line plots shows the mean expression of MYC in CD4+T cell subset from mice on day0 (normal mice), day 7, day 14, and day 21 of EAU modeling. E,F). The basal glycolysis level and glycolysis capacity level of CDLN cells from EAU mice and normal mice. Each group contains six mice. Data expressed as mean ± SEM. Significance was determined using unpaired two‐tailed student's t test. ^**^ *p* <0.01, ^****^ *p* <0.0001. G,H) Representative fundus photographs (G) and clinical scores (H) of EAU mice and 2‐DG treated EAU mic (*n* = 6). White arrows mark inflammatory exudation. I,J) Representative hematoxylin and eosin staining plots of eyes (I) and pathological scores (J) of EAU mice and 2‐DG treated EAU mice at day 14 after immunization (*n* = 6). Black arrows mark retinal folding and inflammatory cell infiltration. Scale bars, 20 mm. Data were expressed as mean ± SEM. Significance was evaluated by unpaired two‐tailed Student t‐test. ^****^ *p* <0.0001. K–M) The proportions of CD4+ cells (K), CD4+ IL‐17A+ cells (L) and CD4+ IFN‐γ + T cells (M) infiltrated into the retina in EAU mice and 2‐DG treated EAU mice at day 14 (*n* = 6). N–P) The proportions of CD4+ IL‐17A+ cells (N), CD4+ IFN‐γ + cells (O), and CD4+ Foxp3+ T cells (P) in CDLNs of EAU mice and 2‐DG treated EAU mice at day 14 (*n* = 6). Data were expressed as mean ± SEM. Significance was evaluated by unpaired two‐tailed Student t‐test. ^****^ *p* <0.0001. ns, not significant.

EAU model was developed to further investigate the role of MYC. Cervical draining lymph nodes (CDLNs) are the major draining lymph nodes of the eyes and may be important sources of auto‐reactive immune cells that target the eyes.^[^ ^44^, ^45^ ^]^ Therefore, we integrated the scRNA‐seq data of CDLN cells from normal mice (day 0 group) and EAU mice on days 7 (day 7 group), 14 (day 14 group), and 21 (day 21 group) (**Figure** **4**A). After initial processing and clustering, eight major immune cells were annotated as previously reported^[^ ^26^ ^]^ (Figure S4A–C, Supporting Information). We first explore the global transcriptional alterations during EAU by conducting DEG analysis on total CDLN cells between normal mice and EAU mice on different days. Compared to normal mice, CDLN cells from EAU mice showed upregulated genes involving antigen processing and presentation (H2‐Aa and H2‐Ab1), inflammation (Fos, Fosb, Nfkb1, S100a11), Th17 cell differentiation (Cebpb), and cell activation (Syk and Slamf6) on different days, while the genes involving in mRNA processing (Srsf7, Sfpq, and Snrnp70) and cell adhesion (Brd2, Skap1, and Thy1) were downregulated Figure S4D, Supporting Information). GO analysis of the upregulated DEGs of CDLN cells in EAU groups showed enrichment in pathways related to antigen processing and presentation, adaptive immune system, positive regulation of immune response, lymphocyte activation, Th17 cell differentiation, cytokine production, and PI3K‐AKT signaling (Figure S4E, Supporting Information). The downregulated DEGs were enriched in pathways related to cell adhesion and mRNA processes (Figure S4F, Supporting Information). We then explored the glycolytic activity of the total CDLN cells. In the total immune cells of CDLNs, GSEA and AUC scores indicated consistent upregulation of the glycolysis pathway on days 7, 14, and 21 compared with day 0 (Figure S4G,H, Supporting Information). The Compass algorithm predicted that during EAU, glycolysis‐associated metabolites were always at higher levels than those in normal mice (Figure S4I–K, Supporting Information).

Subsequently, T cells were re‐clustered and eight T cell subsets were annotated (Figure 4B; Figure S5A,B, Supporting Information). DEG analysis was conducted on T cells between normal mice and EAU mice on different days. T cells showed upregulated genes involving in inflammation (S100a11, Fos, Fosb, Nfkb1, and S100a10), cell activation (Icos), Th17 cell differentiation (Cebpb), and IL‐17 signaling (Junb), as well as downregulated genes involving in cell adhesion (Brd2) and mRNA processing (Sfpq, Rbm38, Pcbp1, Srsf7, and Pcbp1) on different days (Figure S5C, Supporting Information). In addition, Myc also upregulated in T cells of EAU mice on all three time points (Figure S5C, Supporting Information). GO analysis of the upregulated DEGs of T cells in EAU groups showed enrichment in pathways related to TCR signaling, cytokine signaling, IL‐17 signaling, lymphocyte activation, adaptive immune system, Th17 cell differentiation, and PI3K‐AKT signaling (Figure S5D, Supporting Information). The downregulated DEGs of T cells in EAU groups were also enriched in pathways related to cell adhesion and mRNA processes (Figure S5E, Supporting Information). A similar metabolic analysis of T cells indicated a consistently higher glycolytic activity on days 7, 14, and 21 of EAU (Figure 4C; Figures S5F and S6A–C, Supporting Information). Meanwhile, the pathways related to PI3K‐AKT signaling and FOXO signaling were also enhanced in the EAU group (Figure S6D, Supporting Information). We investigated the MYC expression levels in T‐cell subsets. In naïve CD4+ T, Th1, and Th17 cells, MYC expression increased with time after EAU development (Figure 4D). We validated the glycolytic activity of CDLN cells using a seahorse extracellular flux analyzer and found that the basal glycolysis and glycolytic capacity of CDLN cells from EAU mice were higher than those from normal mice (Figure 4E,F). These results showed enhanced glycolysis and the expression of its potential regulator, MYC, in EAU mice, consistent with the results observed in patients with BU.

### 2.5 Inhibiting Glycolysis Disrupted EAU Development

To identify the role of glycolysis in uveitis, we treated EAU mice with the glycolysis inhibitor, 2‐deoxy‐d‐glucose (2‐DG).^[^ ^46^ ^]^ 2‐DG treatment ameliorated EAU symptoms and decreased clinical and pathological scores (Figure 4G–J). Effector T cells (Th1 and Th17 cells) play important pathogenic roles in EAU, whereas Treg cells repress the autoimmune response.^[^ ^47^ ^]^ To identify the impact of glycolysis on T cell subset distribution, flow cytometry was conducted to analyze retina‐infiltrating cells and CDLN cells of EAU mice treated with or without 2‐DG. 2‐DG treatment reduced the proportion of retina‐infiltrating CD4+ T cells, Th1 and Th17 cells (Figure 4K–M; Figure S7A, Supporting Information). In CDLN cells, 2‐DG treatment decreased the proportions of Th1 and Th17 cells (Figure 4N,O; Figure S7B, Supporting Information). However, the proportion of Treg cells was not significantly altered (Figure 4P). We also conducted in vitro experiments. CDLN cells were isolated from EAU mice and treated with the uveitogenic peptide interphotoreceptor retinoid‐binding protein 1–20 (IRBP1‐20) or IRBP1‐20 plus 2‐DG. Upon stimulation with IRBP1‐20, the proportions of Th1 and Th17 cells increased, whereas the proportion of Treg cells decreased (Figure S7C–H, Supporting Information). The enhanced proportion of Th1 and Th17 cells was repressed by 2‐DG treatment; however, it did not alter the proportion of Treg cells (Figure S7C–H, Supporting Information), showing that highly active glycolysis may underlie abnormal Th1 and Th17 responses during EAU. Inhibiting glycolysis may suppress Th1 and Th17 cells and may be a potential therapeutic option for EAU.

### 2.6 Inhibiting MYC Alleviated EAU and Reduced Glycolysis

**Figure 5:**
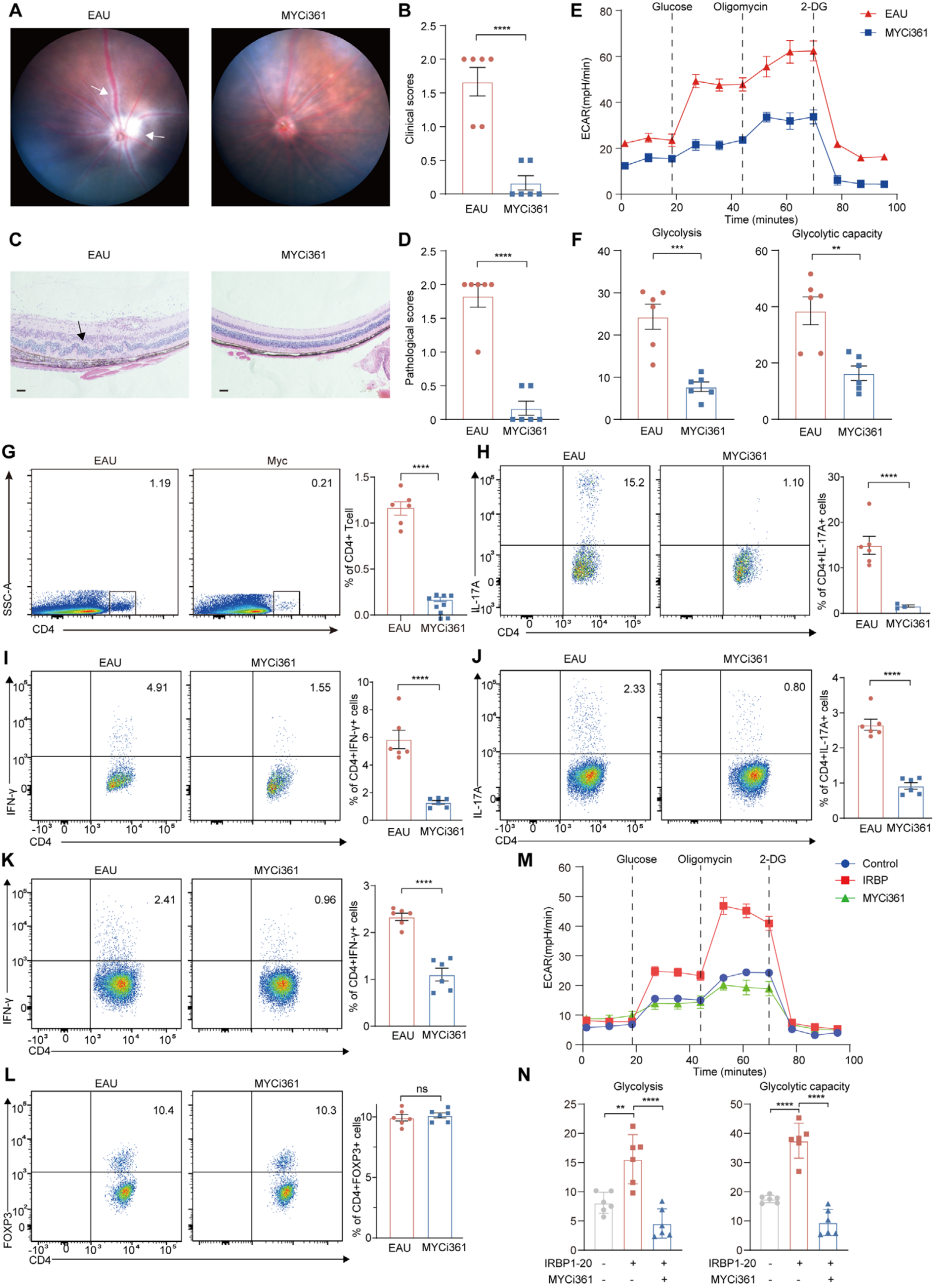
MYCi361 ameliorated EAU and reduced glycolysis. A,B) Representative fundus images (A) and clinical scores (B) of eyes from EAU mice and MYCi361 treated EAU mice at day 14 after immunization. White arrowheads indicate inflammatory exudation (*n* = 6). C,D) Representative hematoxylin and eosin staining images (C) and pathological scores (D) of eyes from EAU mice and MYCi361 treated EAU mice at day 14 after immunization. Black arrowheads indicate infiltration of inflammatory cells and retinal folding (*n* = 6). Data represented as mean ± SEM. Significance was determined using unpaired two‐tailed student's t test. ^****^ *p* <0.0001. Scale bars, 20 mm. E,F) The basal glycolysis level and glycolysis capacity level of CDLN cells from EAU mice and MYCi361 treated EAU mice. Each group contains six mice. Data expressed as mean ± SEM. Significance was determined using unpaired two‐tailed student's t test. **P < 0.01, ***P < 0.001. G–I) The proportions of CD4+ cells (G), CD4+ IL‐17A+ cells (H) and CD4+ IFN‐γ + T cells (I) infiltrated into the retina in EAU mice and MYCi361 treated EAU mice at day 14 after immunization (*n* = 6). Data were expressed as mean ± SEM. Significance was evaluated by unpaired two‐tailed Student t‐test. ^****^ *p* <0.0001. J–L). The proportions of CD4+ IL‐17A+ cells (J), CD4+ IFN‐γ + cells (K), and CD4+ Foxp3+ T cells (L) in CDLNs of EAU mice and MYCi361 treated EAU mice at day 14 after immunization (*n* = 6). Data were expressed as mean ± SEM. Significance was evaluated by unpaired two‐tailed Student t‐test. ^****^ *p* < 0.0001, ns, not significant. M,N) The basal glycolysis level and glycolysis capacity level of isolated CDLN cells treated with IRBP1‐20 or IRBP1‐20 plus MYCi361 for 72 h. Data shown as mean ± SEM of six independent experiments. Data were analyzed using two‐way ANOVA, ^**^ *p* <0.01, ^****^ *p* <0.0001.

To further identify the association between enhanced MYC expression and glycolysis in EAU, mice were treated with MYC inhibitor (MYCi361). MYCi361 treatment reduced the clinical and pathological manifestations of EAU (**Figure** **5**A–D). The glycolytic activity of CDLN cells from EAU mice and MYCi361‐treated‐EAU mice was measured. We found that MYCi361 treatment reduced basal glycolysis and glycolytic capacity in EAU mice (Figure 5E,F). Similar to the effect of glycolysis inhibitors on EAU, the MYC inhibitor reduced the proportion of retina‐infiltrating CD4+ T cells, Th1 and Th17 cells (Figure 5G–I). Moreover, in CDLN cells, the proportions of Th1 and Th17 cells were reduced by MYCi361 (Figure 5J,K). The proportion of Treg cells was not altered by MYCi361 treatment (Figure 5L). These results indicated that MYC may regulate effector T cells by regulating glycolysis during EAU.

### 2.7 MYC Regulated Effector T Cells by Regulating the Glycolysis‐PI3K Signaling Circuit

**Figure 6:**
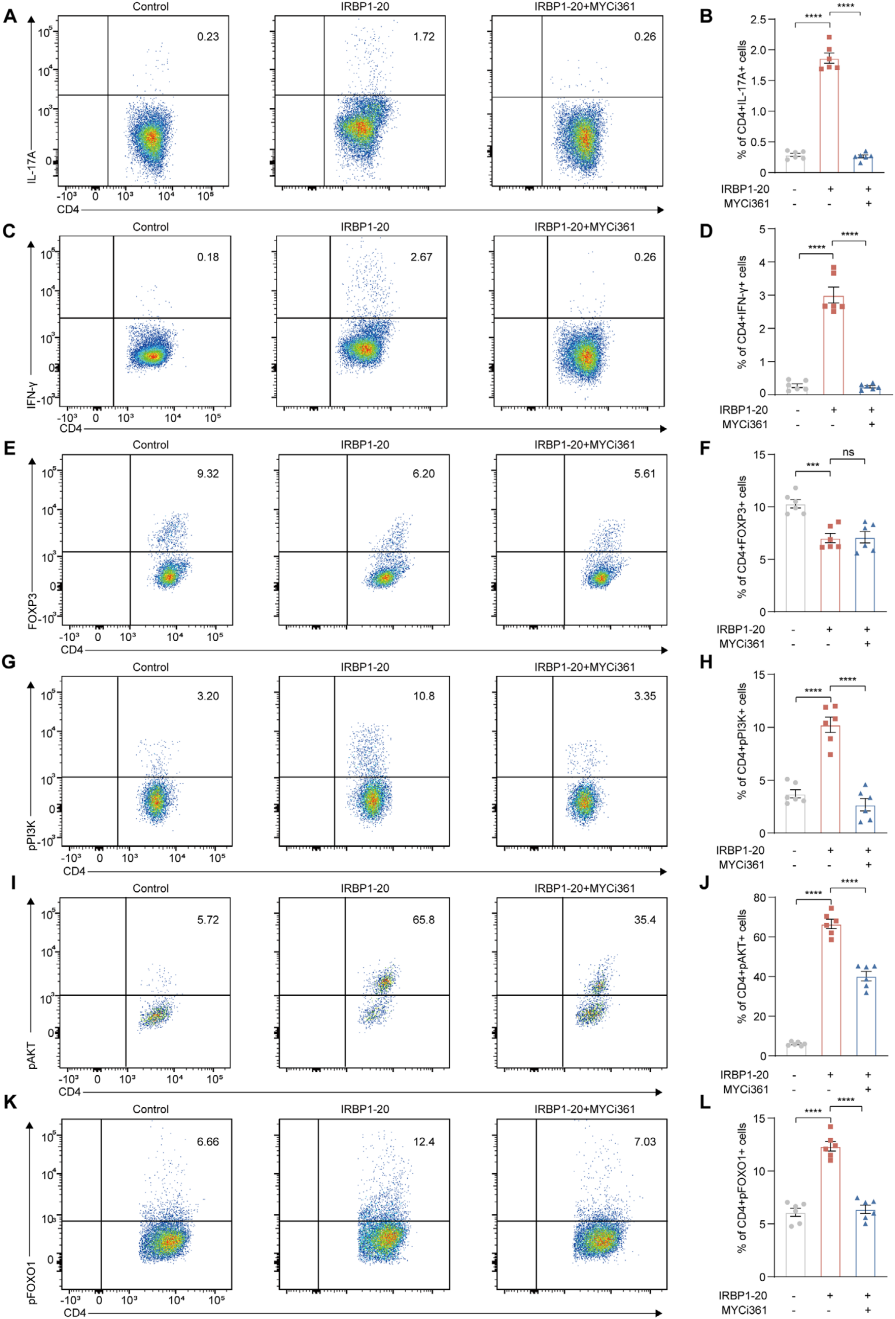
MYCi361 repressed PI3K‐AKT‐FOXO1 pathway. A–F) CDLN cells of EAU mice were collected at day 14 after immunization, treated with IRBP1‐20 or IRBP1‐20 plus MYCi361 for 72 h. The proportions of CD4+ IL‐17A+ cells (A and B), CD4+ IFN‐γ + cells (C and D), CD4+ Foxp3+ cells (E and F) were measured by flow cytometry. Data shown as mean ± SEM of six independent experiments. Data were analyzed using two‐way ANOVA, ^***^ *p* <0.001, ^****^ *p* <0.0001, ns, not significant. G–L) CDLN cells of EAU mice were collected at day 14 after immunization, treated with IRBP1‐20 or IRBP1‐20 plus MYCi361 for 72 h. The proportions of CD4+ pPI3K+ cells (G and H), CD4+ pAKT+ cells (I and J), and CD4+ pFOXO1+ T cells (K and L) were measured by flow cytometry. Data shown as mean ± SEM of six independent experiments. Data were analyzed using two‐way ANOVA, ^****^ *p* <0.0001.

To explore the function of MYC in EAU, in vitro experiments were performed using MYCi361. IRBP1‐20 treatment enhanced glycolysis in isolated CDLN cells, whereas MYCi361 inhibited glycolytic activity (Figure 5M,N). Additionally, MYCi361 treatment offset the promoting effect of IRBP1‐20 on the proportions of Th1 and Th17 cells; however, it did not alter those of Treg cells in vitro (**Figure** **6**A–F), indicating that MYC expression regulated the differentiation of Th1 and Th17 cells by regulating glycolysis. Importantly, CCK‐8 assays showed that MYCi361 did not affect cell viability at the concentration used, indicating that the observed effects were not due to nonspecific cytotoxicity (Figure S7I, Supporting Information). To validate the role of MYC expression in CD4+ T cells in EAU pathogenesis, naïve mice were transferred with autoreactive CD4+ T cells treated with or without MYCi361. MYCi361 treatment impaired the EAU‐inducing ability of CD4+ T‐cells (Figure S7J–M, Supporting Information).

The PI3K‐AKT‐FOXO1 pathway can bolster the effector T cell response.^[^ ^48^, ^49^ ^]^ Additionally, a glycolysis‐PI3K signaling circuit exists in which glycolytic ATP sustains the PI3K signaling, and simultaneously, PI3K signaling induces key glycolytic enzyme expression.^[^ ^22^, ^23^ ^]^ We conducted flow cytometry to explore the effect of MYC expression on the PI3K‐AKT‐FOXO1 pathway in uveitis. IRBP1‐20 treatment stimulated the phosphorylation of PI3K, AKT, and FOXO1 in CD4+ T cells, whereas MYCi361 treatment reversed these changes (Figure 6G–L). Consistent results were obtained using MYC‐targeting short hairpin RNA (MYC shRNA), which also reduced the phosphorylation levels of these signaling molecules in CD4⁺ T cells (Figure S8A–F, Supporting Information). These results indicated that MYC might control the effector T cell response in uveitis by regulating the collaboration of glycolysis and the PI3K‐AKT‐FOXO1 pathway. Inhibiting MYC expression can disrupt the glycolysis‐PI3K signaling circuit to curb abnormal effector T‐cell responses.

## 3 Discussion

In this study, we used untargeted metabolomics and scRNA‐seq to show metabolic signatures and transcriptional alterations in BU. Using untargeted metabolomics and a seahorse extracellular flux analyzer, our study showed that patients with BU had altered serum metabolic profiles and upregulated glycolysis compared to those with HCs. scRNA‐seq data showed that genes involved in adaptive immunity and T‐cell responses were upregulated in BU. Metabolic analysis tools plugged into the scRNA‐seq indicated the enhanced glycolytic capacity of total immune cells and T cell subsets, particularly CD4+Tem cells. Using CytoHubba, MYC was predicted to be a hub gene. Notably, MYC expression was correlated with disease stage and might reflect the treatment response in BU disease. Further experiments based on EAU showed that MYC regulates the effector T cell response in uveitis by regulating the collaboration between PI3K‐AKT‐FOXO1 signaling and glycolysis. MYC inhibitor disrupted the glycolysis‐PI3K signaling circuit to repress abnormal effector T‐cell responses.

Metabolism regulates cellular proliferation, differentiation, and function under healthy and disease conditions.^[^ ^50^, ^51^, ^52^ ^]^ Metabolomics is an “omics” that measures the metabolites produced from various cellular processes using complex statistical and analytical methods.^[^ ^53^ ^]^ This “omics” can reflect the instantaneous physiological and pathological alterations in living organisms.^[^ ^53^ ^]^ Under pro‐inflammatory stimuli, some immune cells undergo a metabolic switch to glycolysis to acquire sufficient energy to support a subsequent immune response.^[^ ^25^ ^]^ Effector T cells, particularly Th1 and Th17 cells, depend on glycolysis for differentiation and effector functions.^[^ ^25^ ^]^ However, the metabolic signature of BU remains unidentified. Our study observed enhanced glycolysis in PBMC from patients with BU and in CDLN cells from EAU mice. Metabolic analysis based on scRNA‐seq data showed activated glycolysis in T cell subsets, particularly in the CD4+Tem cells of patients with BU. Meanwhile, Our analysis revealed that the glycolysis pathway was enhanced in BD without uveitis compared to HC. It has been reported that the glycolysis pathway was elevated in many diseases including arthritis, colitis, non‐alcoholic fatty liver disease, and acute airway inflammation.^[^ ^54^, ^55^, ^56^, ^57^ ^]^ Inhibiting glycolysis can alleviate symptoms of these diseases.^[^ ^54^, ^55^, ^56^, ^57^ ^]^ These findings indicate that the enhancement of glycolysis occurs not only in BU but also in BD without uveitis and other autoimmune inflammatory diseases. Importantly, when using BD without uveitis as disease control, we observed an upregulation of the glycolysis pathway in patients with BU compared to those with BD without uveitis, suggesting a potential involvement of increased glycolysis in the occurrence of uveitis in patients with BD.

Targeting immunometabolism is a therapeutic strategy for treating autoimmune diseases.^[^ ^25^ ^]^ In this study, using 2‐DG, we observed that inhibition of glycolysis ameliorated EAU symptoms and disturbed the effector T cell (Th1 and Th17 cells) response in EAU without affecting the proportion of Treg cells. These results are consistent with the metabolic signature of T cell subsets, in which Th1 and Th17 cells mainly depend on glycolysis,^[^ ^25^ ^]^ while Treg cells oxidize fatty acids for fuel.^[^ ^58^ ^]^ Of the upregulated genes in T cells, CytoHubba predicted MYC to be one of the hub genes that regulate the T cell response in BU. MYC is a proto‐oncogenic transcription factor that binds to DNA to facilitate gene expression.^[^ ^59^, ^60^ ^]^ Dysregulation of MYC is a common occurrence in cancer.^[^ ^61^ ^]^ MYC regulates the transcription of a number of genes involved in multiple cellular processes such as cell proliferation, angiogenesis, programmed cell death, and cellular metabolism.^[^ ^59^, ^62^ ^]^ For instance, MYC can bind to the VEGFA promoter, leading to increased VEGFA production and subsequent sprouting angiogenesis.^[^ ^63^ ^]^ Moreover, MYC is responsible for activating the transcription of CDK4, which supports cell‐cycle progression.^[^ ^64^ ^]^ Additionally, MYC is involved in cellular metabolism. MYC has been reported to promote glycolysis by stimulating the expression of multiple glycolytic enzymes such as hexokinase 2 and pyruvate kinase M2.^[^ ^40^, ^65^ ^]^ Our study explicated the regulatory role of MYC in T cell response in uveitis. MYC promotes glycolysis by inducing the expression of glycolytic enzymes.^[^ ^40^ ^]^ Thus, MYC may regulate T cell responses, particularly those of CD4+ effector T cells, during BU by regulating glycolysis. Using the EAU model, our study showed that the MYC inhibitor strongly restricts glycolytic activities and exerts an effect similar to that of a glycolytic inhibitor (repressing Th1 and Th17 responses and ameliorating EAU symptoms), indicating that MYC regulates the response of CD4 + T effector cells by regulating glycolysis. We further explored how MYC and glycolysis regulate effector T‐cell responses. PI3K‐AKT‐FOXO1 bolsters Th1 and Th17 response by supporting their critical transcriptional factors, T‐bet and RORγt.^[^ ^48^, ^49^ ^]^ Non‐phosphorylated FOXO1 is an inhibitor of T‐bet and RORγt.^[^ ^48^, ^49^ ^]^ PI3K‐AKT signaling phosphorylates FOXO1 to disrupt its inhibitory effects on T‐bet and RORγt.^[^ ^48^, ^49^ ^]^ Recently, several studies have shown that glycolytic ATP is required to fuel PI3K signaling. At the same time, PI3K signaling induces glycolytic enzymes and promotes glycolysis.^[^ ^22^, ^23^ ^]^ These interacting molecules form a positive feedback loop to support metabolic reprogramming of the effector T cell response.^[^ ^22^, ^23^ ^]^ In our study, we confirmed that MYC inhibitors reduced the phosphorylation of PI3K‐AKT‐FOXO1 signaling. Thus, our study indicated that MYC controls the effector T cell response in uveitis by regulating the glycolysis‐PI3K positive feedback circuit. Importantly, the expression of MYC was higher in patients with BU and decreased after effective therapy, while maintaining a high level in patients with inadequate treatment response. These results indicated that MYC might be a potential predictor for the treatment response in BU. However, due to the limited number of sequencing samples, the current findings are suitable for discovery‐phase research. Validation in larger, independent cohorts will be essential in future studies.

## 4 Conclusion

In summary, our study integrates metabolomics and scRNA‐seq to depict an immunometabolicm landscape of BU and found the characteristic metabolic alteration of BU, enhanced glycolysis, as well as the core regulator, MYC. We further observed that the expression of MYC was correlated with BU development and reflected the treatment response. Thus, MYC might be a predictor of treatment response and a novel therapeutic target for patients with BU especially those response inadequately to conventionally therapy.

## 5 Experimental Section

### Mice

Wild‐type C57BL/6J mice (six‐ to eight‐week‐old, 18–25 g) were purchased from Guangdong Medical Lab Animal Center (Guangzhou, China). The mice were housed individually in cages with unrestricted access to food and water under specific pathogen‐free (SPF) conditions. They were maintained at a temperature of 22–24 °C, a relative humidity of 50–70%, and a 12‐h light/dark cycle in the animal facilities at at the Experimental Animal Center of Zhongshan Ophthalmic Center of Sun Yat‐Sen University. All experimental procedures involving mice were approved by the Ethics Committee of Zhongshan Ophthalmic Center of Sun Yat‐Sen University (approval no. 2018‐0189).

### Human Donors

BU was diagnosed based on disease manifestations and the results of standard coherent optical tomography, according to the International Criteria for BD (ICBD).^[^ ^66^ ^]^ Serum from 20 donors diagnosed BU patients and 20 healthy controls were acquired for metabolomic profiling analysis. Blood samples were collected from 6 donors diagnosed with BU and 6 healthy controls for scRNA‐seq. Subsequently, in order to verify the level of glycolysis, another 9 patients with BU and 9 healthy controls were recurited. The above patients had newly onset BU and did not receive any therapy. In addition, the above patients have similar affected organs (uveitis and oral ulcer) and grading for anterior chamber cells and anterior chamber flare (graded as 2+–3+) (Table S1, Supporting Information). Patients with neurological manifestations, vascular manifestations (thrombosis/phlebitis), articular involvement (arthralgia/arthritis/ankylosing spondylitis), or gastrointestinal involvement (diarrhea/proctorrhagia) were excluded.

Additionally, 10 BU patients with improvement after treatment (BU treated) and 10 BU patients who responded inadequately to corticosteroids and immunosuppressive drugs (BU response inadequately) were recruited. The “BU treated” was diagnosed by a two‐step decrease in level of inflammation (anterior chamber cells, vitreous haze) or anterior chamber cell grade of 0.5+ to 0, a vitreous haze grade of 0.5+ to 0, and no newly enlarged active inflammatory choroidal or retinal lesions in both eyes after therapy.^[^ ^67^, ^68^ ^]^ “BU response inadequately” was diagnosed by two‐step increase in the level of inflammation (anterior chamber cells, vitreous haze) or increase from grade 3+ to 4+ according to SUN criteria as well as enlargement active inflammatory choroidal or retinal lesions.^[^ ^67^ ^]^

The clinical information including gender, age, affected organs, disease score, as well as grading for anterior chamber cells and anterior chamber flare was supplied in Table S1 (Supporting Information). No remarkable sex or age differences were observed between patients with BU and healthy controls. All protocols were reviewed and approved by the Medical Ethics Committee (ID:2020KYPJ124). Written informed consent was obtained from all human donors.

### Establishment of EAU

IRBP1‐20 and complete Freund's adjuvant containing Mycobacterium tuberculosis strain H37Ra were used to establish EAU mice. In addition, pertussis toxin in PBS was intraperitoneally injected on day 0 and day 2 after immunization.^[^ ^69^ ^]^ Funduscopic examination of mice and HE staining of the eyeball were performed to grade the clinical scores and the pathological scores as previously described.^[^ ^69^ ^]^

### Treatment Protocols

The mice were intraperitoneally injected daily with 2‐deoxy‐d‐glucose (2‐DG) (50 mg kg^−1^ day^−1^; Selleck Chemicals) or vehicle control for 2 weeks after immunization.^[^ ^20^ ^]^ For the in vitro experiments, cells of cervical draining lymph nodes (CDLNs) were incubated with IRBP1‐20 (20 ug mL^−1^) plus 5 mm 2‐DG or not for 72 h.^[^ ^70^ ^]^

Mice were intraperitoneally injected with MYCi361 (50 mg kg^−1^ day^−1^; Selleck Chemicals) or vehicle control for 2 weeks after immunization.^[^ ^71^ ^]^ For the in vitro experiments, Cells of CDLNs were incubated with IRBP1‐20 (20 ug mL^−1^) plus 5 µm MYCi361 or not for 72 h.^[^ ^71^ ^]^

### Adoptive Transfer Experiment

Cells from the CDLNs of EAU mice (day 14) stimulated with IRBP1‐20 (20 ug mL^−1^) with/without MYCi361 for 72 h, and CD4+ T cells were sorted. Then, the above CD4+ T cells (2 × 10^7^ living cells/mice) were injected intravenously into wild‐type mice.

### Lentivirus Transduction

MYC expression was knocked down using shRNA carried on a lentivirus vector (OBIO, Shanghai, China). The shRNA target sequences for MYC were 5′‐ACGTCTTGGAACGTCAGAG‐3′. The negative control shRNA sequence was 5′‐TTCTCCGAACGTGTCACGT‐3′. Cells were incubated with lentivirus and polybrene (5 µg mL^−1^) for 24 h. Puromycin (5 µg mL^−1^) was added to the culture medium to select transduced cells.

### Flow Cytometry

After staining with live/dead dye, the harvested cells of mice were stained with surface markers. For mice samples, the cells were stained with PerCP/Cyanine5.5‐anti‐CD4 (Biolegend, 100434, 0.2 µg mL); For human samples, the cells were stained with APC‐anti‐CD4 (Biolegend, 344613, 0.2 µg mL^−1^).

For intracellular staining, the cells of mice were stimulated with 50 ng mL^−1^ phorbol myristate acetate (Sigma–Aldrich, St. Louis, MO, USA), 1 µg mL^−1^ brefeldin A (Sigma–Aldrich), and 500 ng mL^−1^ ionomycin (Sigma‐Aldrich) at 37 °C for 4 h. After stimulation, the harvested cells of mice were fixed, permeabilized, stained with Alexa Fluor 647‐anti‐IL‐17A (Biolegend, 506911, 0.3 µg/mL^−1^), PE‐anti‐IFN‐γ (Biolegend, 505808, 0.3 µg mL^−1^), FITC‐anti‐Foxp3 (Invitrogen, 11‐5773‐82, 0.3 µg mL^−1^), APC‐anti‐phospho‐AKT1 (Invitrogen, 17‐9715‐42, 0.5 µg mL^−1^), anti‐phospho‐FOXO1 (Invitrogen, PA5‐104977, 1:100), and anti‐rabbit IgG (H + L), F(ab’)2 Fragment (Alexa Fluor 488 Conjugate) secondary antibody (Cell Signaling Technology, Danvers, MA, USA, 4412, 1:1000). As for intracellular staining with human samples, the harvested cells were fixed, permeabilized, and stained with anti‐MYC (Invitrogen, MA1‐980, 1:100) and anti‐rabbit IgG (H + L), F(ab’)2 Fragment (Alexa Fluor 488 Conjugate) secondary antibody (Cell Signaling Technology, 4412, 1:1000). The cells were analyzed using flow cytometer and the results were analyzed using FlowJo software version 10.0.7 (BD Biosciences).

### CFSE Assay

PBMC from 6 BU patients were collected using Ficoll‐Hypaque solution (GE Healthcare, Chicago, IL, USA), followed by standard density gradient centrifugation. ≈1 × 10^6 CD4+ T cells of PBMC from each BU patient were isolated using flow cytometry. Purified CD4+ T cells were conjugated with 2.5 mm CFSE (BD Biosciences) for 10 min at 37 °C while mixing continuously. Chilled complete growth media was added to quench the labeling reaction. Subsequently, 2 × 10^5 cells labeled with CFSE were incubated with anti‐CD3/CD28 Dynabeads (GIBCO, Grand Island, NY, USA) or anti‐CD3/CD28 Dynabeads plus MYCi361. After incubation for 72 h, the proliferation rate of CD4+ T cells was measured using flow cytometry.

### Seahorse Assay

Seahorse Bioscience XFe24 Extracellular Flux Analyzer (Agilent) was used to measure the glycolytic activity of intact PBMCs and cells of CDLNs by monitoring the ECAR according to the instrument manual. In detail, the XFe24 sensor cartridge was soaked in Calibrant overnight. PBMCs from HC and patients with BU (5 × 10^5 cells per well) or CDLN cells from EAU mice with/without treatment (5 × 10^5 cells per well) were seeded in poly‐lysine coated XFe24‐well seahorse culture microplate in 200 µL of Agilent Seahorse XF RPMI, pH 7.4 containing 2 mm glutamax (Thermofisher). The plate was centrifuged for 60 s at 400 RPM. Three hundred microliters of Agilent Seahorse XF RPMI media were added to the seahorse culture microplate and kept at a CO2‐free incubator at 37 °C for degassing. Injection of glucose as fuel via the first port induced the ECAR levels in cells. The basal glycolytic activity was calculated as the difference between the ECAR reading following the pre‐injection and the injection of glucose. Blanks without cells were automatically subtracted during analysis by Agilent Seahorse XF technology. ECAR values were normalized to cell number (Normalized Rate (well) = [Rate (well)/(Normalization Value)]*Normalization Scale Factor, the Normalization Value = live cell numbers, the Normalization Scale Factor = 100 000).

### Metabolomic Analysis—Sample preparation

Plasma contains a substantial amount of proteins, lipids, and other non‐target components, which can affect the stability of instrument detection and potentially lead to biases that may interfere with metabolite signals. Thus the plasma samples were processed and obtained their extracts for analysis. The collected plasma samples were thawed on ice. Added 400 µL pre‐cooled methanol to 1.5 mL EP tubes. A 100 µL sample was added to the tube and vortexed. The mixture was then stored at −20 °C for 2 h. After centrifuging at 20 000 g for 10 min, the supernatants were transferred into a new 1.5 mL EP tube and dried off. The dried plasma extract for subsequent analysis was stored at −80 °C. Additionally, a pooled extract was prepared for QC by combining 10 µL of extract from each sample.

### Metabolomic Analysis—Liquid Chromatography‐Mass Spectrometry

All samples were analyzed using the Waters ACQUITY UPLC System with an ACQUITY UPLC T3 column (100 mm × 2.1 mm, 1.8 µm). The mobile phase consisted of phase A (5 mmol L^−1^ ammonium acetate + 5 mmol L^−1^ acetic acid in water) and phase B (acetonitrile) under a gradient elution program. Each sample injection volume was 4 µL. A high‐resolution tandem mass spectrometer (TripleTOF 6600, SCIEX) operated in both positive and negative ion modes was used to detect metabolites.

### Metabolomic Analysis—Quality Control and Quality Assurance (QA)

To minimize batch‐to‐batch variability, all clinical samples were processed and analyzed in a single analytical batch. Specifically, sample preparation, extraction, and LC‐MS analysis were conducted in a continuous sequence without inter‐batch interruptions. Additionally, during the sequencing process, the samples were analyzed in a random order to avoid potential grouping effects based on sample type or acquisition time (Table S4, Supporting Information).

To monitor and correct for potential signal drift and ensure system stability, a total of 7 QC samples were inserted at regular intervals throughout the sequence: three QC samples were used initially to stabilize the system, and one QC sample was run after every 10 experimental samples.

### Metabolomic Analysis—Batch Effect Evaluation and Correction

Although all samples were processed and analyzed in a single analytical batch, potential intra‐batch effects were carefully evaluated using unsupervised principal component analysis (PCA). PCA plots demonstrated no significant clustering based on injection order or other potential confounding factors, confirming the absence of detectable batch effects. Furthermore, quality control‐based robust LOESS signal correction was applied to QC samples to minimize any potential signal drift.

### Metabolomic Analysis—Data Preprocessing and Normalization

The intensity of peak data was further preprocessed by metaX. Those features that were detected in less than 50% of QC samples or 80% of biological samples were removed, and the remaining peaks with missing values were imputed with the k‐nearest neighbor algorithm to further improve the data quality. PCA was performed for outlier detection and batch effects evaluation using the pre‐processed dataset. Probabilistic Quotient Normalization (PQN) was used to normalize the data to obtain the normalized ion intensity data of each sample. In addition, the coefficient of variation (CV) of the metabolic features was calculated across all QC samples, and those >30% were then removed.

### Metabolomic Analysis—Metabolites identification

The acquired MS data pretreatments including peak picking, peak grouping, retention time correction, second peak grouping, as well as annotation of isotopes and adducts were performed using XCMS software. LC−MS raw data files were converted into mzXML format and then processed by the XCMS, CAMERA, and metaX toolbox implemented with the R software. Each ion was identified by combining retention time (RT) and m/z data. Intensities of each peak were recorded and a 3D matrix containing arbitrarily assigned peak indices (retention time‐m/z pairs), sample names (observations), and ion intensity information (variables) was generated.

The online HMDB database was used to annotate the metabolites of samples by matching the exact molecular mass data, name, and formula of samples with those from the database. If a mass difference between the observed and the database value was less than 10 ppm, the metabolite would be annotated and the molecular formula of metabolites would be further identified and validated by the isotopic distribution measurements. Compound mass spectrometry databases and an in‐house fragment spectrum library of metabolites were also used to validate the metabolite identification. Thus, metabolites were annotate by matching MS1 using the online HMDB database and matching MS/MS spectra using compound mass spectrometry databases, both in‐house and open source. According to the Metabolomics Standards Initiative (MSI),^[^ ^21^ ^]^ the confidence level for this identification is level 2. Only metabolites of level 2 would be used for subsequent analysis.

### Metabolomic Analysis—KEGG Enrichment Analysis

Supervised PLS‐DA was conducted to discriminate the different variables between groups, and the VIP value was calculated. A VIP cut‐off value of 1.0 was used to select important features. In the multivariate models, seven‐fold cross‐validation was used to safeguard against overfitting. Non‐parametric methods (Mann‐Whitney U test) were conducted to detect significantly differences in metabolite between the 2 groups (p value adjusted by FDR <0.05). KEGG enrichment analysis was performed on significantly different metabolites.^[^ ^20^ ^]^

### Isolation of CDLNs and PBMC for scRNA‐Seq

For pipeline analysis, CDLN cells were harvested from normal group and EAU groups at different time points. The cell viability in each sample exceeded 85%. For PBMC, the venous blood samples were extracted using Ficoll‐Hypaque solution, then processed via standard density gradient centrifugation approaches to obtain PBMCs. The cell viability was more than 85% in all the samples.

### scRNA‐Seq Data Processing

scRNA‐seq libraries were generated using the Chromium Single Cell 5′ Library and Gel Bead Kit (10x Genomics, 120237) according to the manufacturer's instructions. In detail, after washing with 0.04% BSA buffer (0.02 g BSA dissolved in 50 mL of deionized PBS), cells were captured in droplets. Then, reverse transcription, emulsion breaking, barcoded‐cDNA purification with Dynabeads, and PCR amplification were conducted step by step. The amplified cDNA was then used to construct the 5′ gene expression library. Specifically, fragmenting and end‐repair, double‐size selection with SPRIselect beads, and sequencing were conducted on 50 ng of amplified cDNA using the NovaSeq platform (Illumina NovaSeq6000) to yield 150 bp paired‐end reads. Initial processing of the sequenced data was performed using CellRanger software v3.0.2 (10x Genomics). Data were analyzed using the Scanpy package (v1.9.1) in python (v3.7.12). The batch effect across different samples was removed using the bbknn package (v1.1.5).

### Dimensionality Reduction and Clustering Analysis

For scRNA‐seq data analysis with Scanpy, Scanpy used “pp.normalize_total” to scale and standardize the count values of the single‐cell expression matrix, commonly known as CPM (counts per million). Furtherly, in order to better balance the impact of different sequencing depths among different samples, Scanpy further used the “pp. log1p” function to perform log (CPM+1) conversion on CPM values. After that, “sc.tl.leiden” was used to cluster cells, and the “sc.pl.umap” function was used to visualize with a 2D UMAP algorithm. Additionally, “sc.tl.rank_genes” was used to generate marker genes of different clusters and differentially expressed genes (DEGs) on distinct cell types. To understand the global transcriptional changes during BU, DEG analysis between all immune cells from the BU group and those from the HC group was conducted using “sc.tl.rank_genes” of Scanpy. Genes with | Log2 (fold change) | >0.5 and adjusted *p* values <0.05 were considered as DEGs and used for further analysis. The “sc pl.violin” was used to generate violin plots to show the differential expression of specific genes between the patients with BU and the healthy controls. Additionally, “compass –gene expression matrix –species homo_sapiens/mus_musculus” of Compass and “sc.metabolism” of scMetabolism are used to infer changes in cellular metabolic states.

### GO Analysis

Gene ontology (GO) analysis was performed using the Metascape web tool (www.metascape.org). Among the top 50 enriched GO terms across different cell types, 5–10 GO terms or pathways that were associated with diseases were shown.

### Protein‐Protein Interaction Network Construction and Hub Genes Prediction

The upregulated DEGs of T cells in the BU/HC comparison group were inputted into the String database^[^ ^38^ ^]^ to construct the protein‐protein interaction network with a 0.4 (medium confidence) minimum required interaction score. The output results were visualized by Cytascape (v3.9.1). Subsequently, the CytoHubba tool^[^ ^39^ ^]^ in Cytoscape was used (applying 10 different algorithms including Betweenness, Stress, Radiality, MNC, MCC, EPC, EcCentricity, Degree, Closeness, and BottleNeck) to predict the top 20 core genes respectively in the protein‐protein interaction network.

### Statistics

All experiments were repeated at least three times. GraphPad Prism Software, version 8.0.2 (GraphPad, Inc., La Jolla, CA, USA) was employed for data analysis and presentation. The results are presented as the means ± SEM.

### Ethics Approval and Consent to Participate

All protocols involving human data were reviewed and approved by the Medical Ethics Committee (ID:2020KYPJ124). Written informed consent was obtained from all human donors. In addition, all experiments involving mice were approved by the Ethics Committee of Zhongshan Ophthalmic Center of Sun Yat‐Sen University (approval no. 2018‐0189).

### Availability of Data and Materials

The single‐cell sequencing data generated in this study was deposited in the Genome Sequence Archive (GSA) under project number (PRJCA021035) and GSA accession number (mouse data: CRA017163; human data: HRA006506). The data analysis pipeline used in the study was described on the 10X Genomics (https://www.10xgenomics.com/) and Scanpy official websites (https://github.com/scverse/scanpy). Compass and scMetabolism are both used to infer changes in cellular metabolic states in scRNA‐seq data analysis. The detailed usage and related code for Compass (https://yoseflab.github.io/Compass/tutorial.html) and scMetabolism (https://github.com/wu‐yc/scMetabolism) have been published on GitHub.

## Conflict of Interest

The authors declare no conflict of interest.

## Author Contributions

H.L., L.Z., Z.L., Y.Z., and G.Z. contributed equally to the work. W. S., S.Z., and R.J. conceived of and supervised the project. W.S. recruited and provided patient care, and clinical and histological assessments. H.L., Y.Z., and L.Z. performed the experiments. Z.L., X.P., X.Z., and S.Z. performed bioinformatic analysis. D.W. and J.C. processed clinical data. R.W., L.Z., Q.J, and S.L. wrote the manuscript. All authors discussed and approved the manuscript.

## Supporting information



Supporting Information

Supplemental Table 1

Supplemental Table 2

Supplemental Table 3

Supplemental Table 4

## Data Availability

The data that support the findings of this study are openly available in the Genome Sequence Archive (GSA) under project number (PRJCA021035) and GSA accession number (mouse data: CRA017163; human data: HRA006506).

## References

[1] M. Joubert , A.‐C. Desbois , F. Domont , A. Ghembaza , A. Le Joncour , A. Mirouse , G. Maalouf , M. Leclercq , S. Touhami , P. Cacoub , B. Bodaghi , D. Saadoun , J. Clin. Med. 2023, 12, 3648. 37297843 10.3390/jcm12113648 PMC10253549

[2] M. D. de Smet , M. Dayan , Invest. Ophthalmol. Vis. Sci. 2000, 41, 3480. 11006242

[3] J. H. Yamamoto , M. Minami , G. Inaba , K. Masuda , M. Mochizuki , Br. J. Ophthalmol. 1993, 77, 584. 8218058 10.1136/bjo.77.9.584 PMC513957

[4] M. Mesquida , B. Molins , V. Llorenç , M. S. de la Maza , M. V. Hernandez , G. Espinosa , A. Adán , Mediators Inflamm. 2014, 2014, 396204. 24994946 10.1155/2014/396204 PMC4068062

[5] A. Greco , A. De Virgilio , M. Ralli , A. Ciofalo , P. Mancini , G. Attanasio , M. de Vincentiis , A. Lambiase , Autoimmun Rev. 2018, 17, 567. 29631062 10.1016/j.autrev.2017.12.006

[6] Y. R. Hsu , J. C. Huang , Y. Tao , T. Kaburaki , C. S. Lee , T. C. Lin , C. C. Hsu , S. H. Chiou , D. K. Hwang , Eye (Lond) 2019, 33, 66. 30323327 10.1038/s41433-018-0223-z PMC6328561

[7] J. Shi , C. Zhao , J. Zhou , J. Liu , L. Wang , F. Gao , X. Zeng , M. Zhang , W. Zheng , Ther. Adv. Chronic Dis. 2019, 10, 2040622319847881. 31105923 10.1177/2040622319847881 PMC6505232

[8] Q. Jiang , Q. Wang , S. Tan , J. Cai , X. Ye , G. Su , P. Yang , Invest. Ophthalmol. Vis. Sci. 2023, 64, 28. 10.1167/iovs.64.4.28 PMC10148662 37093132

[9] F. Ilhan , T. Demir , P. Türkçüoğlu , B. Turgut , N. Demir , A. Gödekmerdan , Can. J. Ophthalmol. 2008, 43, 105. 18204495 10.3129/i07-179

[10] R. R. Caspi , P. B. Silver , D. Luger , J. Tang , L. M. Cortes , G. Pennesi , M. J. Mattapallil , C.‐C. Chan , Ophthalmic Res. 2008, 40, 169. 18421234 10.1159/000119871 PMC2735820

[11] D. Luger , P. B. Silver , J. Tang , D. Cua , Z. Chen , Y. Iwakura , E. P. Bowman , N. M. Sgambellone , C. C. Chan , R. R. Caspi , J. Exp. Med. 2008, 205, 799. 18391061 10.1084/jem.20071258 PMC2292220

[12] Z. Huang , W. Li , W. Su , Adv. Exp. Med. Biol. 2021, 1278, 205. 33523450 10.1007/978-981-15-6407-9_11

[13] Y. Iwasaki , Y. Takeshima , M. Nakano , M. Okubo , M. Ota , A. Suzuki , Y. Kochi , T. Okamura , T. Endo , I. Miki , K. Sakurada , K. Yamamoto , K. Fujio , Rheumatology 2023, 62, 905. 35689621 10.1093/rheumatology/keac338

[14] M. D. Buck , R. T. Sowell , S. M. Kaech , E. L. Pearce , Cell 2017, 169, 570. 28475890 10.1016/j.cell.2017.04.004 PMC5648021

[15] M. Liebmann , S. Hucke , K. Koch , M. Eschborn , J. Ghelman , A. I. Chasan , S. Glander , M. Schädlich , M. Kuhlencord , N. M. Daber , M. Eveslage , M. Beyer , M. Dietrich , P. Albrecht , M. Stoll , K. B. Busch , H. Wiendl , J. Roth , T. Kuhlmann , L. Klotz , Proc. Natl. Acad. Sci. USA 2018, 115, E8017. 30072431 10.1073/pnas.1721049115 PMC6112725

[16] S. K. S. Bhagavatham , P. Khanchandani , V. Kannan , D. Potikuri , D. Sridharan , S. K. Pulukool , A. A. Naik , R. B. Dandamudi , S. M. Divi , A. Pargaonkar , R. Ray , S. S. R. Santha , P. B. Seshagiri , K. Narasimhan , N. Gumdal , V. Sivaramakrishnan , Sci. Rep. 2021, 11, 15129. 34301999 10.1038/s41598-021-94607-5 PMC8302689

[17] A. A. Kolodziejczyk , J. K. Kim , V. Svensson , J. C. Marioni , S. A. Teichmann , Mol. Cell 2015, 58, 610. 26000846 10.1016/j.molcel.2015.04.005

[18] A. Wagner , C. Wang , J. Fessler , D. DeTomaso , J. Avila‐Pacheco , J. Kaminski , S. Zaghouani , E. Christian , P. Thakore , B. Schellhaass , E. Akama‐Garren , K. Pierce , V. Singh , N. Ron‐Harel , V. P. Douglas , L. Bod , A. Schnell , D. Puleston , R. A. Sobel , M. Haigis , E. L. Pearce , M. Soleimani , C. Clish , A. Regev , V. K. Kuchroo , N. Yosef , Cell 2021, 184, 4168. 34216539 10.1016/j.cell.2021.05.045 PMC8621950

[19] Y. Wu , S. Yang , J. Ma , Z. Chen , G. Song , D. Rao , Y. Cheng , S. Huang , Y. Liu , S. Jiang , J. Liu , X. Huang , X. Wang , S. Qiu , J. Xu , R. Xi , F. Bai , J. Zhou , J. Fan , X. Zhang , Q. Gao , Cancer Discov. 2022, 12, 134. 34417225 10.1158/2159-8290.CD-21-0316

[20] I. Zahoor , H. Suhail , I. Datta , M. E. Ahmed , L. M. Poisson , J. Waters , F. Rashid , R. Bin , J. Singh , M. Cerghet , A. Kumar , M. N. Hoda , R. Rattan , A. K. Mangalam , S. Giri , Proc. Natl. Acad. Sci. USA 2022, 119, 2123265119. 10.1073/pnas.2123265119 PMC9231486 35700359

[21] I. Blaženović , T. Kind , J. Ji , O. Fiehn , Metabolites 2018, 8, 31. 29748461 10.3390/metabo8020031 PMC6027441

[22] K. Xu , N. Yin , M. Peng , E. G. Stamatiades , S. Chhangawala , A. Shyu , P. Li , X. Zhang , M. H. Do , K. J. Capistrano , C. Chou , C. S. Leslie , M. O. Li , Immunity 2021, 54, 976. 33979589 10.1016/j.immuni.2021.04.008 PMC8130647

[23] K. Xu , N. Yin , M. Peng , E. G. Stamatiades , A. Shyu , P. Li , X. Zhang , M. H. Do , Z. Wang , K. J. Capistrano , C. Chou , A. G. Levine , A. Y. Rudensky , M. O. Li , Science 2021, 371, 405. 33479154 10.1126/science.abb2683 PMC8380312

[24] R. Noguchi , H. Kubota , K. Yugi , Y. Toyoshima , Y. Komori , T. Soga , S. Kuroda , Mol. Syst. Biol. 2013, 9, 664. 23670537 10.1038/msb.2013.19 PMC4039368

[25] E. M. Pålsson‐McDermott , L. A. J. O'Neill , Cell Res. 2020, 30, 300. 32132672 10.1038/s41422-020-0291-z PMC7118080

[26] H. Li , L. Xie , L. Zhu , Z. Li , R. Wang , X. Liu , Z. Huang , B. Chen , Y. Gao , L. Wei , C. He , R. Ju , Y. Liu , X. Liu , Y. Zheng , W. Su , Nat. Commun. 2022, 13, 5866. 36195600 10.1038/s41467-022-33502-7 PMC9532430

[27] X. Liu , Q. Jiang , J. Lv , S. Yang , Z. Huang , R. Duan , T. Tao , Z. Li , R. Ju , Y. Zheng , W. Su , JCI Insight 2022, 7, 162335. 10.1172/jci.insight.162335 PMC9746911 36301664

[28] L. Zhu , H. Li , R. Wang , Z. Li , S. Zhao , X. Peng , W. Su , Invest. Ophthalmol. Vis. Sci. 2023, 64, 24. 10.1167/iovs.64.5.24 PMC10214869 37227746

[29] C. Zhang , L. Gao , B. Wang , Y. Gao , Brief Bioinform. 2021, 22, bbab147. 33940590 10.1093/bib/bbab147

[30] S. Aibar , C. B. González‐Blas , T. Moerman , V. A. Huynh‐Thu , H. Imrichova , G. Hulselmans , F. Rambow , J.‐C. Marine , P. Geurts , J. Aerts , J. van den Oord , Z. K. Atak , J. Wouters , S. Aerts , Nat. Methods 2017, 14, 1083. 28991892 10.1038/nmeth.4463 PMC5937676

[31] H. Direskeneli , H. Fujita , C. A. Akdis , J. Allergy Clin. Immunol. 2011, 128, 665. 21878243 10.1016/j.jaci.2011.07.008

[32] R. R. Caspi , J. Clin. Invest. 2010, 120, 3073. 20811163 10.1172/JCI42440 PMC2929721

[33] W. P. Chong , M. J. Mattapallil , K. Raychaudhuri , S. J. Bing , S. Wu , Y. Zhong , W. Wang , Z. Chen , P. B. Silver , Y. Jittayasothorn , C.‐C. Chan , J. Chen , R. Horai , R. R. Caspi , Immunity 2020, 53, 384. 32673565 10.1016/j.immuni.2020.06.022 PMC7362799

[34] H. Li , L. Zhu , R. Wang , L. Xie , J. Ren , S. Ma , W. Zhang , X. Liu , Z. Huang , B. Chen , Z. Li , H. Feng , G.‐H. Liu , S. Wang , J. Qu , W. Su , Protein Cell 2022, 13, 422. 34748200 10.1007/s13238-021-00882-3 PMC9095810

[35] S. Liu , S. Liao , L. Liang , J. Deng , Y. Zhou , Trends Endocrinol. Metab. 2023, 34, 345. 37061430 10.1016/j.tem.2023.03.006

[36] C.‐H. Chang , J. D. Curtis , L. B. Maggi , B. Faubert , A. V. Villarino , D. O'Sullivan , S. C.‐C. Huang , G. J. W. van der Windt , J. Blagih , J. Qiu , J. D. Weber , E. J. Pearce , R. G. Jones , E. L. Pearce , Cell 2013, 153, 1239. 23746840 10.1016/j.cell.2013.05.016 PMC3804311

[37] Z. Wu , Y. Zheng , J. Sheng , Y. Han , Y. Yang , H. Pan , J. Yao , Front. Immunol. 2022, 13, 816005. 35222392 10.3389/fimmu.2022.816005 PMC8866817

[38] D. Szklarczyk , J. H. Morris , H. Cook , M. Kuhn , S. Wyder , M. Simonovic , A. Santos , N. T. Doncheva , A. Roth , P. Bork , L. J. Jensen , C. von Mering , Nucleic Acids Res. 2017, 45, D362. 27924014 10.1093/nar/gkw937 PMC5210637

[39] C. H. Chin , S. H. Chen , H. H. Wu , C. W. Ho , M. T. Ko , C. Y. Lin , BMC Syst. Biol. 2014, 8, S11. 25521941 10.1186/1752-0509-8-S4-S11 PMC4290687

[40] C. V. Dang , A. Le , P. Gao , Clin. Cancer Res. 2009, 15, 6479. 19861459 10.1158/1078-0432.CCR-09-0889 PMC2783410

[41] A. Amadi‐Obi , C. R. Yu , X. Liu , R. M. Mahdi , G. L. Clarke , R. B. Nussenblatt , I. Gery , Y. S. Lee , Nat. Med. 2007, 13, 711. 17496900 10.1038/nm1585

[42] Y. Deng , Y. Zhang , T. Cai , Q. Wang , W. Zhang , Z. Chen , X. Luo , G. Su , P. Yang , J. Autoimmun. 2022, 133, 102920. 36191467 10.1016/j.jaut.2022.102920

[43] W. Zheng , X. Wang , J. Liu , X. Yu , L. Li , H. Wang , J. Yu , X. Pei , C. Li , Z. Wang , M. Zhang , X. Zeng , F. Zhang , C. Wang , H. Chen , H.‐Z. Chen , Proc. Natl. Acad. Sci. U SA 2022, 119, 2204289119. 10.1073/pnas.2204289119 PMC9245671 35727985

[44] Y. H. Yücel , K. Cardinell , S. Khattak , X. Zhou , M. Lapinski , F. Cheng , N. Gupta , Invest. Ophthalmol. Vis. Sci. 2018, 59, 2699. 29860456 10.1167/iovs.17-22850

[45] J. Grüntzig , H. Schicha , F. Huth , Z. Lymphol. 1979, 3, 35. 231868

[46] Z. Zheng , H. Ma , X. Zhang , F. Tu , X. Wang , T. Ha , M. Fan , L. Liu , J. Xu , K. Yu , R. Wang , J. Kalbfleisch , R. Kao , D. Williams , C. Li , J. Infect. Dis. 2017, 215, 1396. 28368517 10.1093/infdis/jix138 PMC5451607

[47] S. J. Bing , I. Shemesh , W. P. Chong , R. Horai , Y. Jittayasothorn , P. B. Silver , B. Sredni , R. R. Caspi , J. Autoimmun. 2019, 100, 52. 30853312 10.1016/j.jaut.2019.02.006 PMC6513711

[48] A. Lainé , B. Martin , M. Luka , L. Mir , C. Auffray , B. Lucas , G. Bismuth , C. Charvet , J. Immunol. 2015, 195, 1791. 26170390 10.4049/jimmunol.1500849

[49] E. E. Kraus , L. Kakuk‐Atkins , M. F. Farinas , M. Jeffers , A. E. Lovett‐Racke , Y. Yang , J. Neuroimmunol. 2021, 359, 577675. 34403862 10.1016/j.jneuroim.2021.577675 PMC8435019

[50] M. G. Vander Heiden , L. C. Cantley , C. B. Thompson , Science 2009, 324, 1029. 19460998 10.1126/science.1160809 PMC2849637

[51] L. Sun , X. Yang , Z. Yuan , H. Wang , Arterioscler. Thromb. Vasc. Biol. 2020, 40, 1990.32698683

[52] T. Gaber , C. Strehl , F. Buttgereit , Nat. Rev. Rheumatol. 2017, 13, 267. 28331208 10.1038/nrrheum.2017.37

[53] A. H. Zhang , H. Sun , X. J. Wang , Anal. Bioanal. Chem. 2013, 405, 8143. 23715678 10.1007/s00216-013-7061-4

[54] R. Garcia‐Carbonell , A. S. Divakaruni , A. Lodi , I. Vicente‐Suarez , A. Saha , H. Cheroutre , G. R. Boss , S. Tiziani , A. N. Murphy , M. Guma , Arthritis Rheumatol. 2016, 68, 1614. 26815411 10.1002/art.39608 PMC4963240

[55] S. M. Hochrein , H. Wu , M. Eckstein , L. Arrigoni , J. S. Herman , F. Schumacher , C. Gerecke , M. Rosenfeldt , D. Grün , B. Kleuser , G. Gasteiger , W. Kastenmüller , B. Ghesquière , J. Van den Bossche , E. D. Abel , M. Vaeth , Cell Metab. 2022, 34, 516. 35316657 10.1016/j.cmet.2022.02.015 PMC9019065

[56] M. E. Moreno‐Fernandez , D. A. Giles , J. R. Oates , C. C. Chan , M. S. M. A. Damen , J. R. Doll , T. E. Stankiewicz , X. Chen , K. Chetal , R. Karns , M. T. Weirauch , L. Romick‐Rosendale , S. A. Xanthakos , R. Sheridan , S. Szabo , A. S. Shah , M. A. Helmrath , T. H. Inge , H. Deshmukh , N. Salomonis , S. Divanovic , Cell Metab. 2021, 33, 1187. 34004162 10.1016/j.cmet.2021.04.018 PMC8237408

[57] J. Q. Yang , K. W. Kalim , Y. Li , S. Zhang , A. Hinge , M. D. Filippi , Y. Zheng , F. Guo , J. Allergy Clin. Immunol. 2016, 137, 231. 26100081 10.1016/j.jaci.2015.05.004 PMC4684821

[58] J. A. Maciolek , J. A. Pasternak , H. L. Wilson , Curr. Opin. Immunol. 2014, 27, 60. 24556090 10.1016/j.coi.2014.01.006

[59] Z. E. Stine , Z. E. Walton , B. J. Altman , A. L. Hsieh , Cancer Discov. 2015, 5, 1024. 26382145 10.1158/2159-8290.CD-15-0507 PMC4592441

[60] J. Y. Kim , Y. E. Cho , J. H. Park , Am. J. Pathol. 2015, 185, 2061. 25956029 10.1016/j.ajpath.2015.03.016

[61] R. Dhanasekaran , A. Deutzmann , W. D. Mahauad‐Fernandez , A. S. Hansen , A. M. Gouw , D. W. Felsher , Nat. Rev. Clin. Oncol. 2022, 19, 23. 34508258 10.1038/s41571-021-00549-2 PMC9083341

[62] S. E. Ahmadi , S. Rahimi , B. Zarandi , R. Chegeni , M. Safa , J. Hematol. Oncol. 2021, 14, 121. 34372899 10.1186/s13045-021-01111-4 PMC8351444

[63] Y. Shi , X. Xu , Q. Zhang , G. Fu , Z. Mo , G. S. Wang , S. Kishi , X. L. Yang , Elife 2014, 3, 02349. 10.7554/eLife.02349 PMC4057782 24940000

[64] H. Hermeking , C. Rago , M. Schuhmacher , Q. Li , J. F. Barrett , A. J. Obaya , B. C. O'Connell , M. K. Mateyak , W. Tam , F. Kohlhuber , C. V. Dang , J. M. Sedivy , D. Eick , B. Vogelstein , K. W. Kinzler , Proc. Natl. Acad. Sci. USA 2000, 97, 2229. 10688915 10.1073/pnas.050586197 PMC15783

[65] C. J. David , M. Chen , M. Assanah , P. Canoll , J. L. Manley , Nature 2010, 463, 364. 20010808 10.1038/nature08697 PMC2950088

[66] F. Davatchi , S. Assaad‐Khalil , K. T. Calamia , J. E. Crook , B. Sadeghi‐Abdollahi , M. Schirmer , T. Tzellos , C. C. Zouboulis , M. Akhlagi , A. Al‐Dalaan , Z. S. Alekberova , A. A. Ali , A. Altenburg , E. Arromdee , M. Baltaci , M. Bastos , S. Benamour , I. Ben Ghorbel , A. Boyvat , L. Carvalho , W. Chen , E. Ben‐Chetrit , C. Chams‐Davatchi , J. A. Correia , J. Crespo , C. Dias , Y. Dong , F. Paixão‐Duarte , K. Elmuntaser , A. V. Elonakov , et al., J. Eur. Acad. Dermatol. Venereol. 2014, 28, 338. 23441863 10.1111/jdv.12107

[67] D. A. Jabs , R. B. Nussenblatt , J. T. Rosenbaum , Am. J. Ophthalmol. 2005, 140, 509. 16196117 10.1016/j.ajo.2005.03.057 PMC8935739

[68] Z. Zhong , G. Su , P. Yang , Prog. Retin. Eye Res. 2023, 97, 101216. 37734442 10.1016/j.preteyeres.2023.101216

[69] R. K. Agarwal , P. B. Silver , R. R. Caspi , Methods Mol. Biol. 2012, 900, 443. 22933083 10.1007/978-1-60761-720-4_22 PMC3810964

[70] Y. Yin , S. C. Choi , Z. Xu , D. J. Perry , H. Seay , B. P. Croker , E. S. Sobel , T. M. Brusko , L. Morel , Sci. Transl. Med. 2015, 7, 274ra218. 10.1126/scitranslmed.aaa0835 PMC5292723 25673763

[71] H. Han , A. D. Jain , M. I. Truica , J. Izquierdo‐Ferrer , J. F. Anker , B. Lysy , V. Sagar , Y. Luan , Z. R. Chalmers , K. Unno , H. Mok , R. Vatapalli , Y. A. Yoo , Y. Rodriguez , I. Kandela , J. B. Parker , D. Chakravarti , R. K. Mishra , G. E. Schiltz , S. A. Abdulkadir , Cancer Cell 2019, 36, 483. 31679823 10.1016/j.ccell.2019.10.001 PMC6939458

