# Supplementary material for: Integrated Transcriptome and Metabolomics Analyses Show MYC as a Potential Therapeutic Target for Behçet's Uveitis
Source: Adv Sci (Weinh). 2025 Jun 30;12(36):e17843. doi: 10.1002/advs.202417843 (PMC12463086; doi:10.1002/advs.202417843)
Supplement: Supplementary file 1 — Supporting Information [file ADVS-12-e17843-s002.docx]

**Supplementary Figures and Figure legends**

**Figure S1**


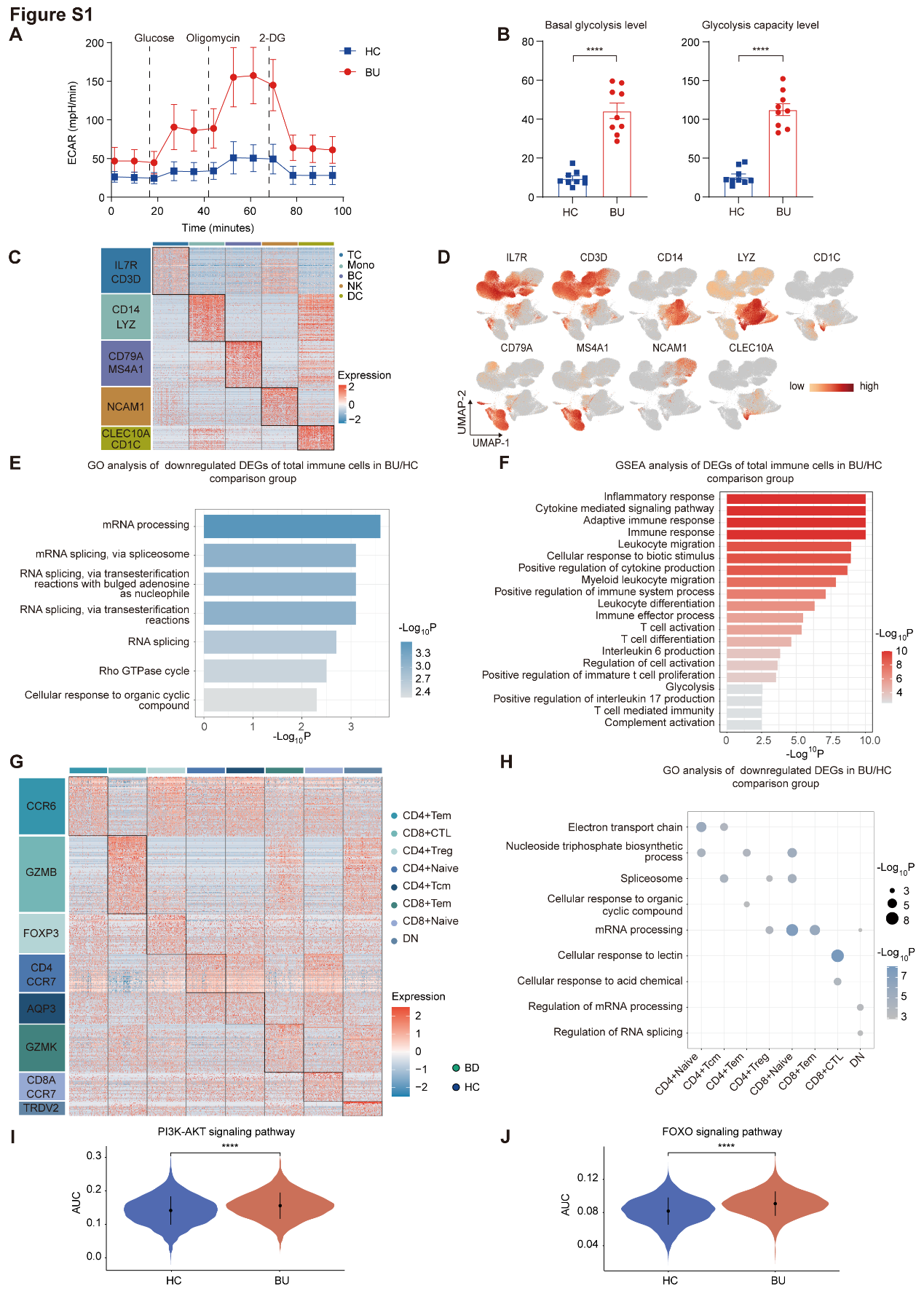


**Supplementary Figure.1**

A-B. The basal glycolysis level and glycolysis capacity level of CD4+ T cells from patients with BU and HCs were measured by glycolysis stress assay. Each group contains nine samples. Data expressed as mean ± SEM. Significance was determined using unpaired two-tailed student’s t test. ****P < 0.0001.

C. Heatmap shows scaled expression of discriminative gene sets for the major immune cell types of PBMC from all participants.

D. Feature plots show the expression of discriminative gene sets for the major immune cell types.

E. Bar plot shows representative GO terms enriched by the downregulated DEGs of PBMCs from patients with BU compared to HCs. The color represents enrichment P value.

F. Bar plot shows representative GSEA terms enriched by the DEGs of PBMCs from patients with BU compared to HCs. The color represents enrichment P value.

G. Heatmap shows scaled expression of discriminative gene sets for T cell subsets of all participants.

H. Representative GO terms enriched by the downregulated DEGs of each T cell subset from patients with BU compared to HCs. The circle size and color represent enrichment P value.

I-J. Violin plot shows AUcell score of PI3K-AKT signalling pathway and FOXO signalling pathway in PBMCs from patients with BU and HCs. Significance was evaluated by wilcoxon test. ****P < 0.0001.

**Figure S2**


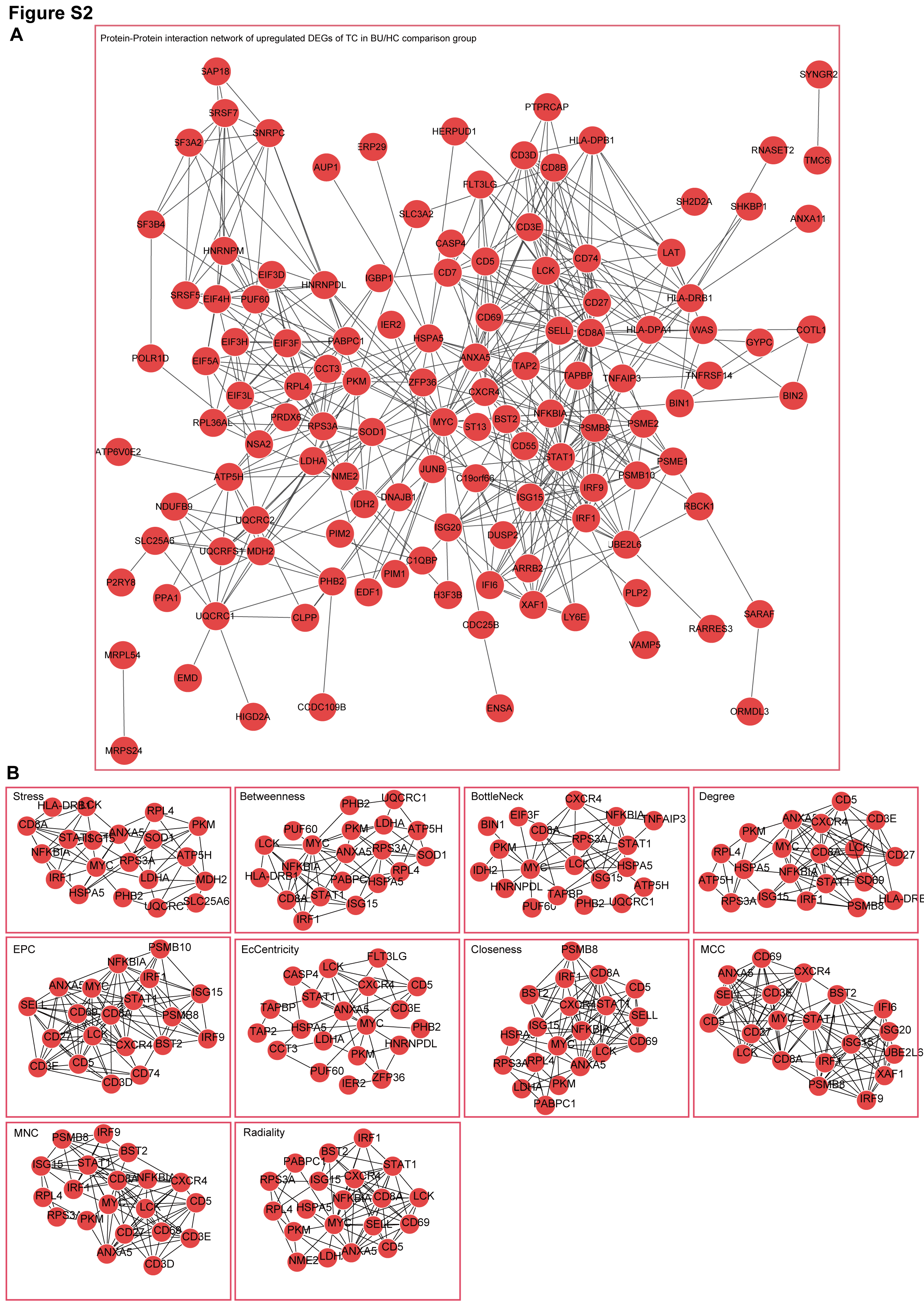


**Supplementary Figure.2**

A. PPI network of upregulated DEGs of T cells.

B. Networks of top 20 hub genes of T cells predicted by different algorithms.

**Figure S3**


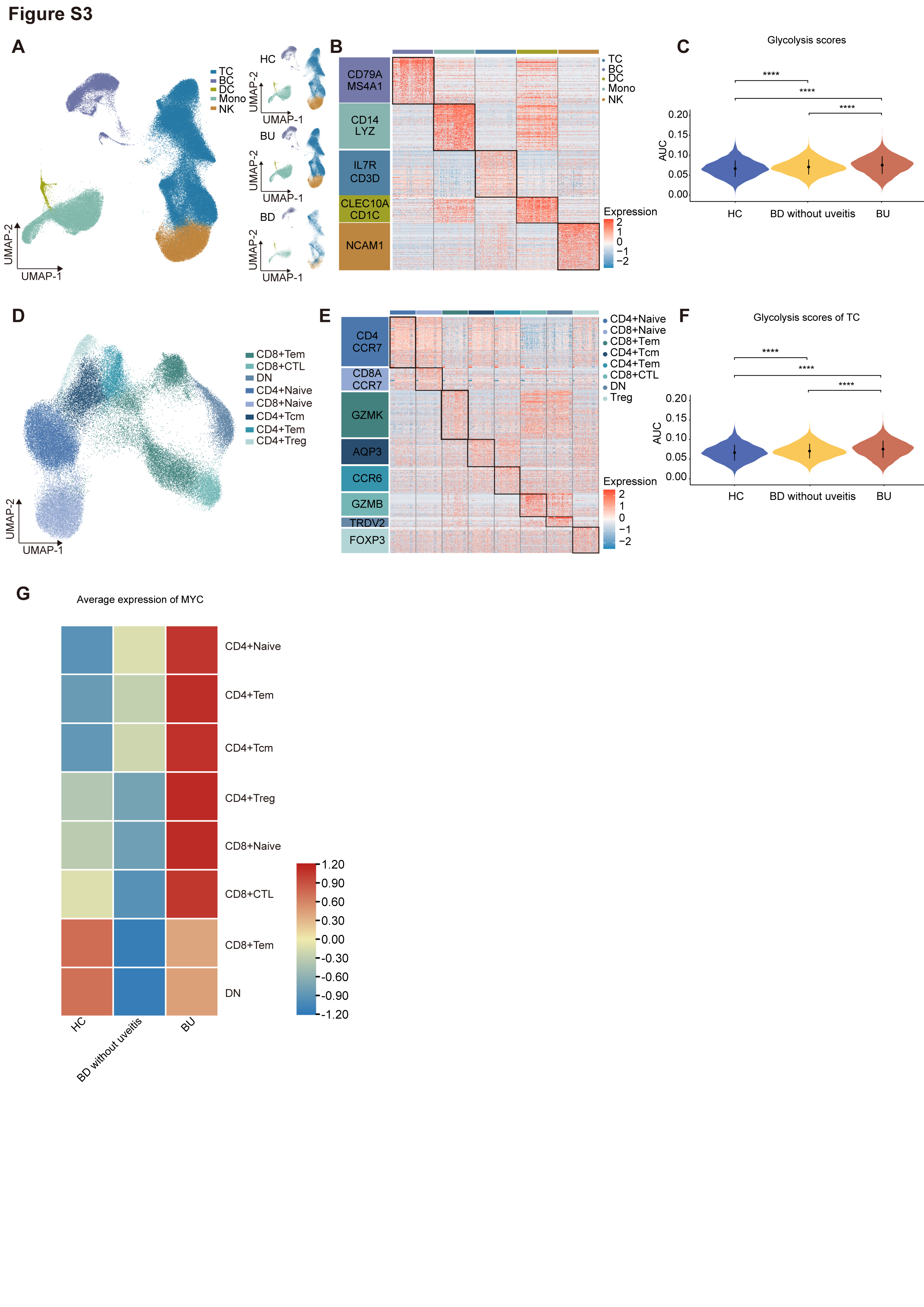


**Supplementary Figure.3**

A. UMAP clustering of PBMCs.

B. Heatmap shows scaled expression of discriminative gene sets for the major immune cell types of PBMCs.

C. Violin plot shows AUcell score of glycolysis in PBMCs from HCs, BD without uveitis and patients with BU. Significance was evaluated by wilcoxon test. ****P < 0.0001.

D. UMAP clustering of T cells.

E. Heatmap shows scaled expression of discriminative gene sets for T cell subsets.

F. Violin plot shows AUcell score of glycolysis in T cells from HCs, BD without uveitis and patients with BU. Significance was evaluated by wilcoxon test. ****P < 0.0001.

G. Heatmap shows average expression of MYC in T cell subsets from HCs, BD without uveitis and patients with BU.

**Figure S4**


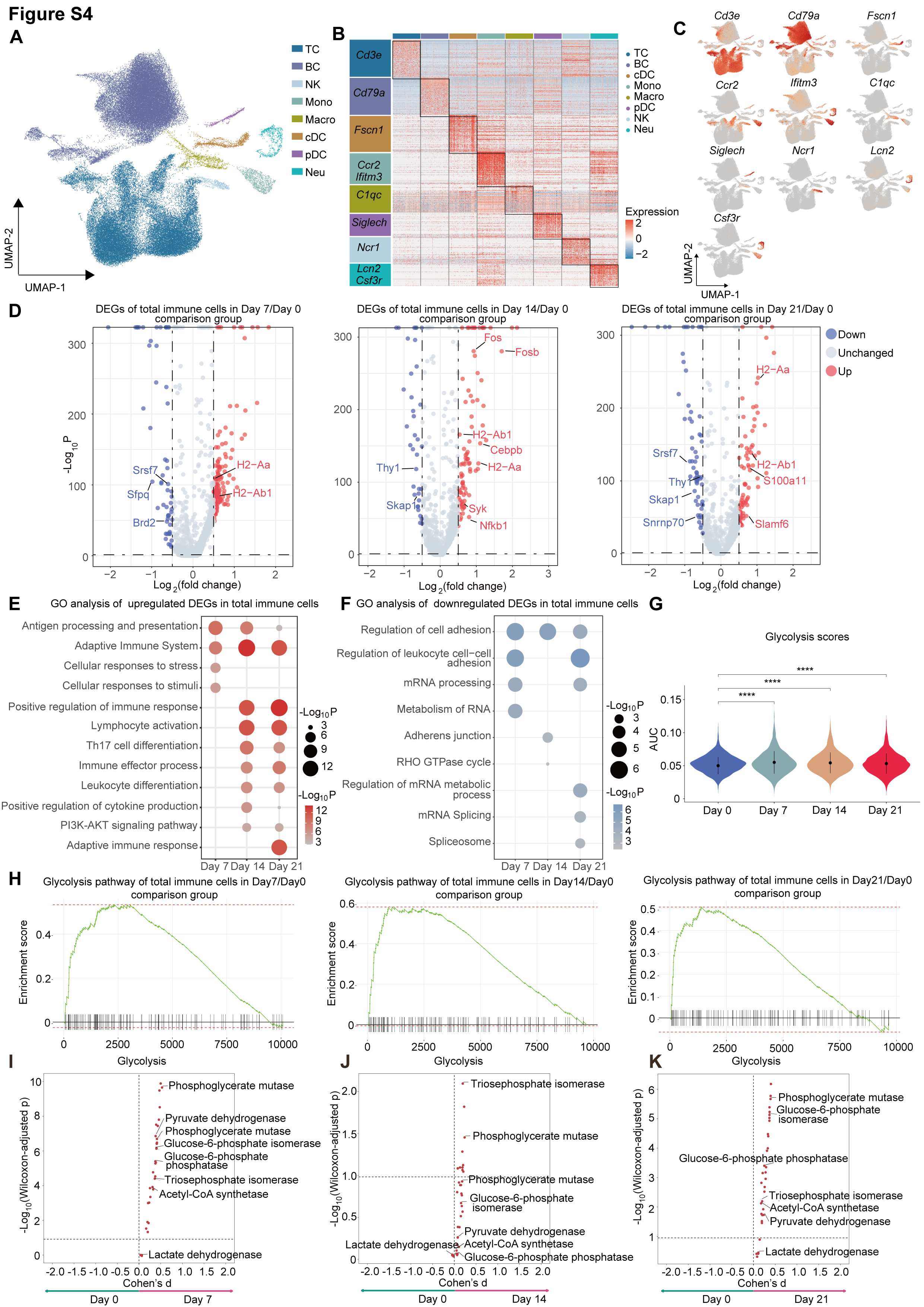


**Supplementary Figure.4**

A. UMAP clustering of CDLN cells.

B. Heatmap shows scaled expression of discriminative gene sets for the major immune cell types.

C. Feature plots shows the expression of discriminative gene sets for the major immune cell types.

D. Volcano plot shows DEGs of CDLN cells between EAU mice (day 7, day 14, and day 21) and normal mice (day 0). Red and blue dots indicate upregulated and downregulated genes respectively.

E-F. Bubble plot shows representative GO terms enriched by the upregulated (E) and downregulated (F) DEGs of CDLN cells from EAU mice (day 7, day 14, and day 21) compared to normal mice (day 0). The circle size and color represent enrichment P value.

G. Violin plot shows AUcell score of glycolysis in CDLN cells. Significance was evaluated by wilcoxon test. ****P < 0.0001.

H. GSEA of DEGs in CDLN cells from mice on day 7, day 14, or day 21 of EAU modeling compared to normal mice (day 0) shows the enrichment of glycolysis pathway.

I-K. Compass-score glycolysis activity in CDLN cells from mice on day 7 (I), day 14 (J), and day 21 (K) of EAU modeling compared to day 0.

**Figure S5**


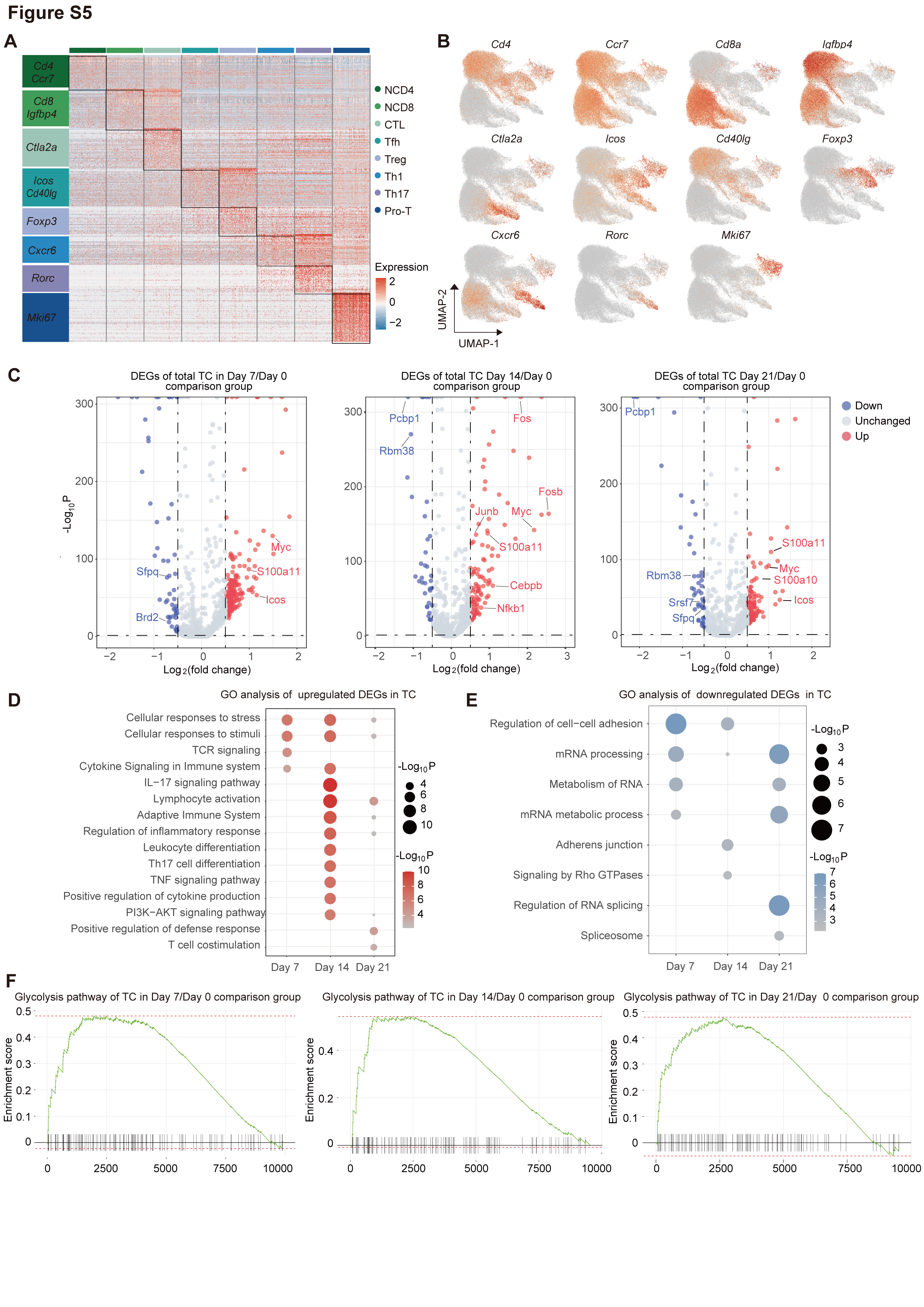


**Supplementary Figure.5**

A. Heatmap shows scaled expression of discriminative gene sets for the T cell subsets.

B. Feature plots shows the expression of discriminative gene sets for the T cell subsets.

C. Volcano plot shows DEGs of T cells between EAU mice (day 7, day 14, and day 21) and normal mice (day 0). Red and blue dots indicate upregulated and downregulated genes respectively

D-E. Bubble plot shows representative GO terms enriched by the upregulated (D) and downregulated (E) DEGs of T cells from EAU mice (day 7, day 14, and day 21) compared to normal mice (day 0). The circle size and color represent enrichment P value.

F. GSEA of DEGs in T cells from mice on day 7, day 14, and day 21 of EAU modeling compared to day 0 (normal mice) shows the enrichment of glycolysis pathway.

**Figure S6**


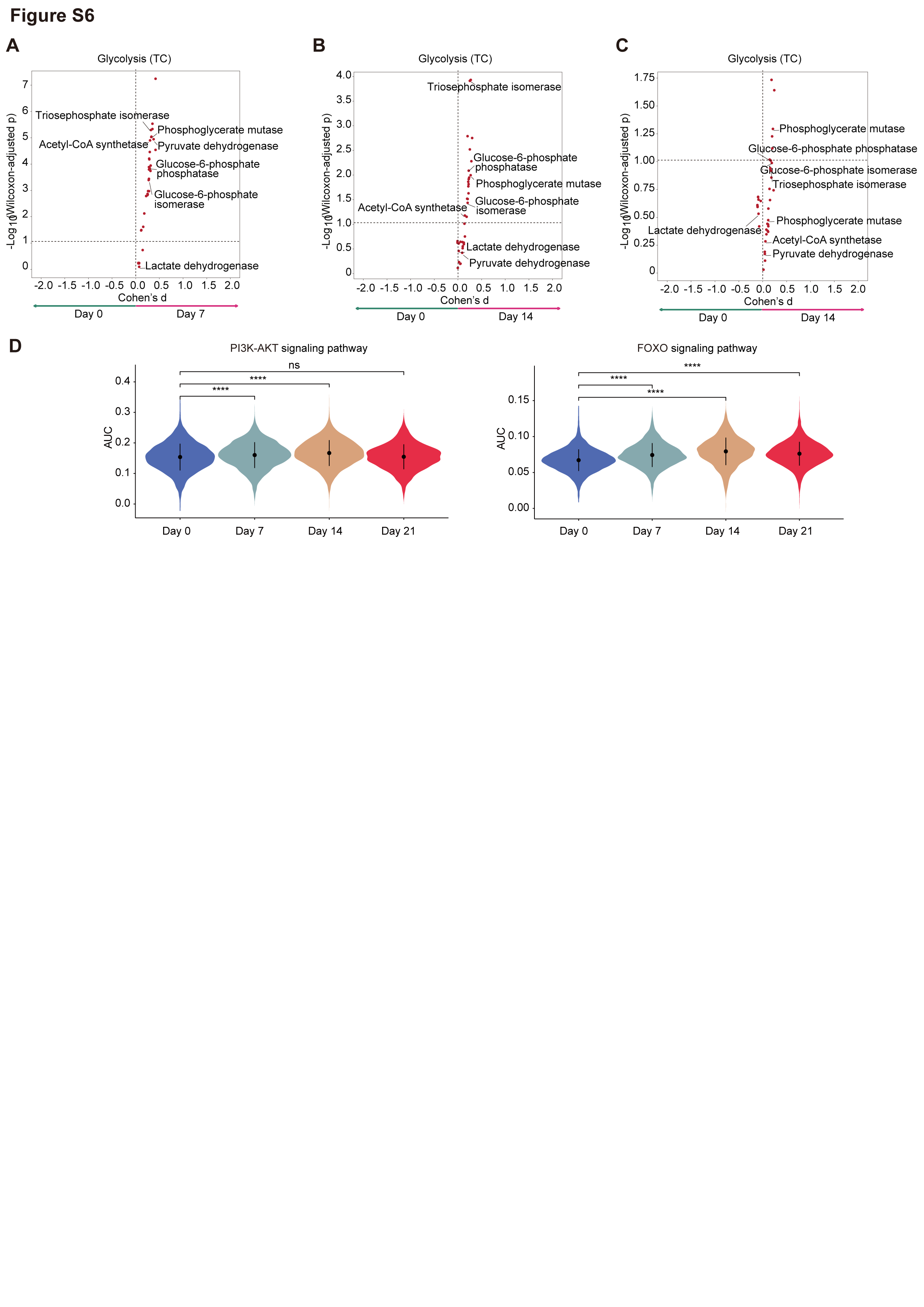


**Supplementary Figure.6**

A-C. Compass-score glycolysis activity in T cells from mice on day 7 (A), day 14 (B), and day 21 (C) of EAU modeling compared to day 0 (normal mice).

D. Violin plot shows AUcell score of PI3K-AKT signalling pathway and FOXO signalling pathway in T cells from mice on day 0 (normal mice), day 7, day 14, and day 21 of EAU modeling. Significance was evaluated by wilcoxon test. ****P < 0.0001. ns, not significant.

**Figure S7**


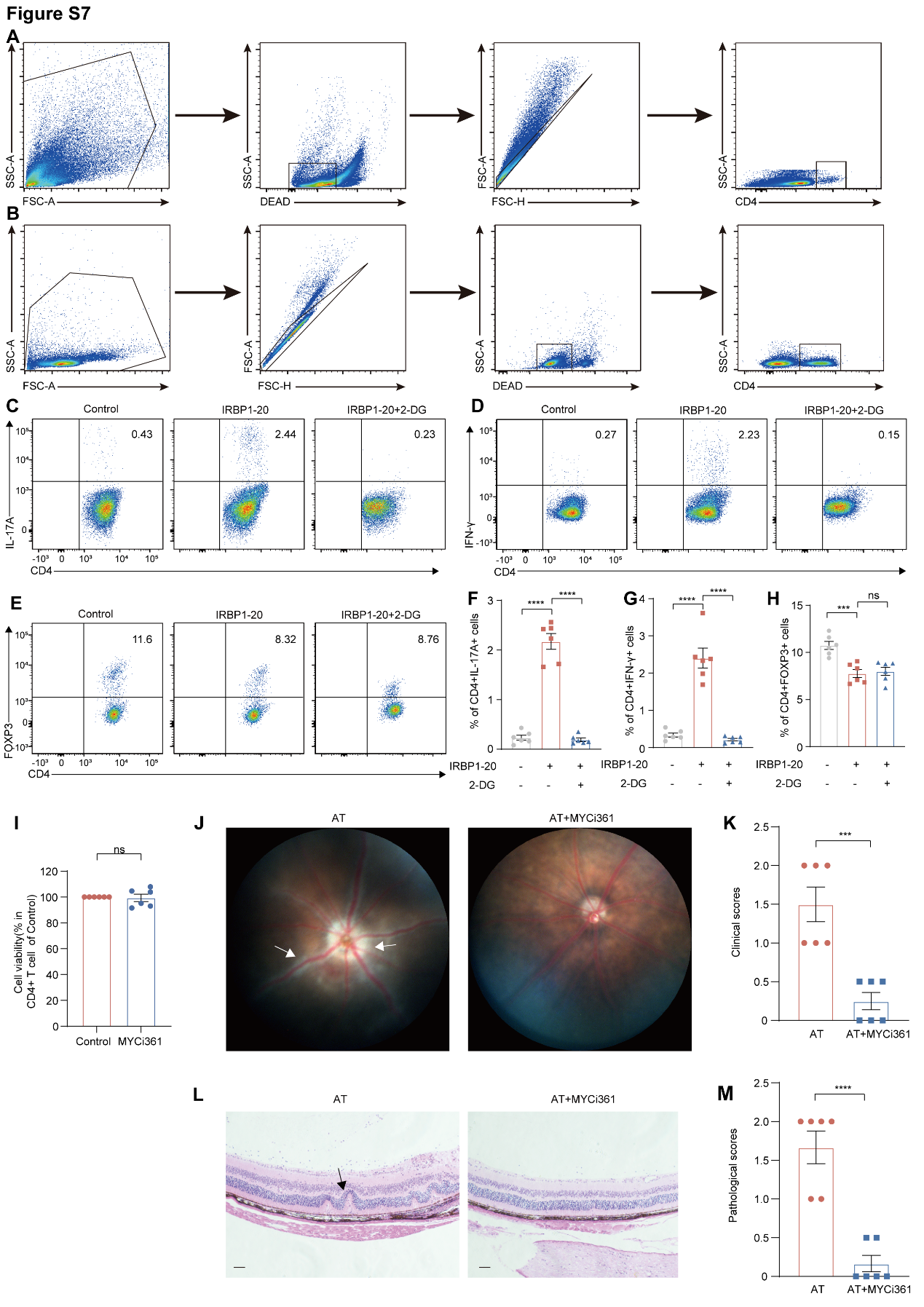


**Supplementary Figure.7**

A. Gating strategy of infiltrated CD4+ T cells in retina.

B. Gating strategy of CD4+ T cells in CDLNs.

C-H. CDLN cells of EAU mice were isolated and treated with IRBP1-20 or IRBP1-20 plus 2-DG for 72 hours. The proportions of Th17 cells (C and F), Th1 cells (D and G), and Treg (E and H) were measured. N=6. Significance was evaluated by two-way ANOVA, ***P < 0.001, ****P < 0.0001, ns, not significant.

I. CCK8 assay of CD4+ T cells isolated from CDLN cells treated by MYCi361 (5 μM) for 72 h. Data was shown as mean ± SEM from six independent experiments. Significance was evaluated by unpaired two-tailed student’s t test. ns, not significant.

J-K. Representative fundus images (J) and clinical scores (K) of eyes from mice injected with CD4+ T cells cultured with IRBP1–20 or IRBP1–20+ MYCi361 at day 14. White arrowheads indicate inflammatory exudation. N=6.

L-M. Representative hematoxylin and eosin staining images (L) and pathological scores (M) of eyes from mice injected with CD4+ T cells cultured with IRBP1–20 or IRBP1–20+ MYCi361 at day 14. Black arrowheads indicate infiltration of inflammatory cells and retinal folding. N=6. Scale bars, 20 mm. AT: Adoptive transfer experiment. Significance was evaluated by unpaired two-tailed student’s t test. ***P < 0.001, ****P < 0.0001.


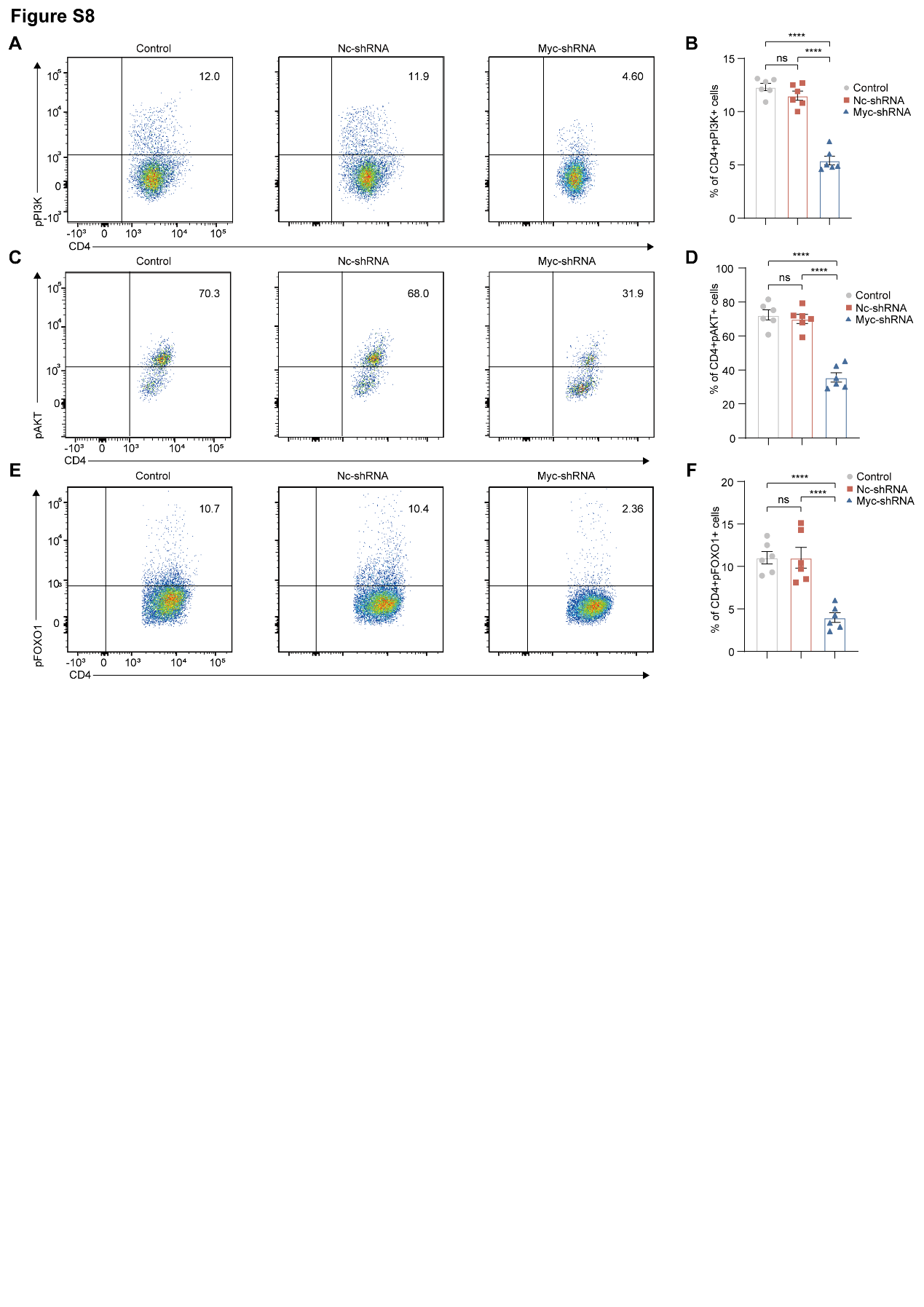


**Supplementary Figure.8**

A-F. After treated with Myc shRNA or negative control (Nc) shRNA, CD4+ T cells from EAU mice were cultured with IRBP1-20 for 72 hours. The proportions of CD4+ pPI3K+ cells (A and B), CD4+ pAKT+ cells (C and D), and CD4+ pFOXO1+ T cells (E and F) were measured by flow cytometry. Data shown as mean ± SEM of six independent experiments. Data were analyzed using two-way ANOVA, ****P < 0.0001.
